# Investigating causality in the association between DNA methylation and type 2 diabetes using bidirectional two-sample Mendelian randomisation

**DOI:** 10.1007/s00125-023-05914-7

**Published:** 2023-05-19

**Authors:** Diana L. Juvinao-Quintero, Gemma C. Sharp, Eleanor C. M. Sanderson, Caroline L. Relton, Hannah R. Elliott

**Affiliations:** 1 MRC Integrative Epidemiology Unit, Bristol Medical School, University of Bristol, Bristol, UK; 2 Population Health Sciences, Bristol Medical School, University of Bristol, Bristol, UK; 3 Department of Epidemiology, Harvard T. H. Chan School of Public Health, Boston, MA USA

**Keywords:** Causality, DNA methylation, Epigenetics, Mendelian randomisation, Type 2 diabetes

## Abstract

**Aims/hypothesis:**

Several studies have identified associations between type 2 diabetes and DNA methylation (DNAm). However, the causal role of these associations remains unclear. This study aimed to provide evidence for a causal relationship between DNAm and type 2 diabetes.

**Methods:**

We used bidirectional two-sample Mendelian randomisation (2SMR) to evaluate causality at 58 CpG sites previously detected in a meta-analysis of epigenome-wide association studies (meta-EWAS) of prevalent type 2 diabetes in European populations. We retrieved genetic proxies for type 2 diabetes and DNAm from the largest genome-wide association study (GWAS) available. We also used data from the Avon Longitudinal Study of Parents and Children (ALSPAC, UK) when associations of interest were not available in the larger datasets. We identified 62 independent SNPs as proxies for type 2 diabetes, and 39 methylation quantitative trait loci as proxies for 30 of the 58 type 2 diabetes-related CpGs. We applied the Bonferroni correction for multiple testing and inferred causality based on *p*<0.001 for the type 2 diabetes to DNAm direction and *p*<0.002 for the opposing DNAm to type 2 diabetes direction in the 2SMR analysis.

**Results:**

We found strong evidence of a causal effect of DNAm at cg25536676 (*DHCR24*) on type 2 diabetes. An increase in transformed residuals of DNAm at this site was associated with a 43% (OR 1.43, 95% CI 1.15, 1.78, *p*=0.001) higher risk of type 2 diabetes. We inferred a likely causal direction for the remaining CpG sites assessed. In silico analyses showed that the CpGs analysed were enriched for expression quantitative trait methylation sites (eQTMs) and for specific traits, dependent on the direction of causality predicted by the 2SMR analysis.

**Conclusions/interpretation:**

We identified one CpG mapping to a gene related to the metabolism of lipids (*DHCR24*) as a novel causal biomarker for risk of type 2 diabetes. CpGs within the same gene region have previously been associated with type 2 diabetes-related traits in observational studies (BMI, waist circumference, HDL-cholesterol, insulin) and in Mendelian randomisation analyses (LDL-cholesterol). Thus, we hypothesise that our candidate CpG in *DHCR24* may be a causal mediator of the association between known modifiable risk factors and type 2 diabetes. Formal causal mediation analysis should be implemented to further validate this assumption.

**Graphical Abstract:**

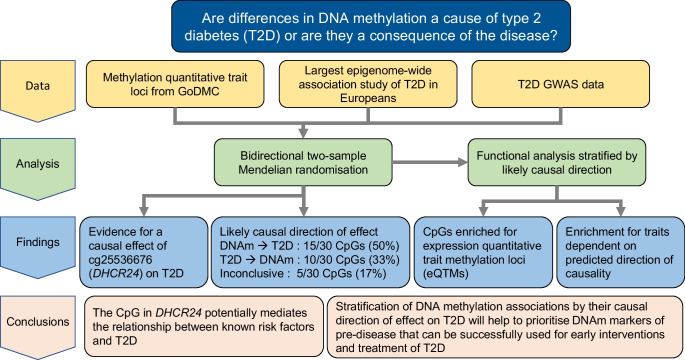

**Supplementary Information:**

The online version of this article (10.1007/s00125-023-05914-7) contains peer-reviewed but unedited supplementary material.

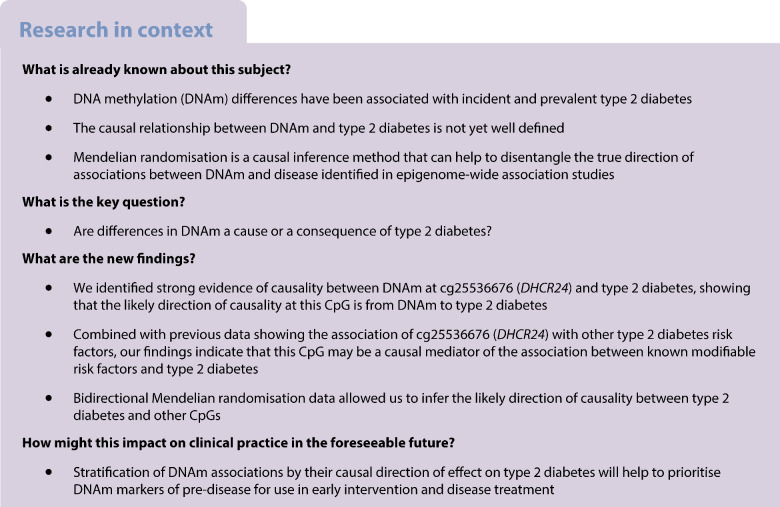



## Introduction

There is growing interest in understanding the role of DNA methylation (DNAm) in the context of type 2 diabetes. In epidemiological studies, associations have been identified between DNAm and both prevalent [1–8] and incident [9–14] type 2 diabetes. Associations between DNAm and type 2 diabetes may arise through different mechanisms. For example, changes in DNAm may be causal for type 2 diabetes. Conversely, type 2 diabetes may induce consequent changes in DNAm. It is also possible that the observed association between DNAm and type 2 diabetes arises as a result of confounding from a third factor that is independently associated with both type 2 diabetes and DNAm. Defining the causal relationship between DNAm and type 2 diabetes is important because it provides new information about the molecular pathways involved in disease incidence, and potentially progression, allowing new insights into targets for intervention. A method that uses genetic variants to estimate the causal direction of effect between a modifiable exposure and an outcome, while controlling for unobserved confounders, is Mendelian randomisation (MR) [15].

Previous studies have investigated the causal direction of effect between DNAm and type 2 diabetes using MR. In the context of incident type 2 diabetes, MR was used to assess the causal link between 18 incident type 2 diabetes-associated CpG sites and type 2 diabetes [11]. Methylation quantitative trait loci (mQTL) were available for 16 of the 18 type 2 diabetes-associated CpG sites. There was nominal evidence for a direct causal association of cg00574958 (*CPT1A*) with type 2 diabetes. The authors of this study also assessed the causal effects of BMI and glycaemic traits on methylation at the 18 CpG sites associated with incident type 2 diabetes but found no evidence to support this causal pathway [11]. In the context of prevalent type 2 diabetes, a study of 232 participants with type 2 diabetes and 197 control participants from a Korean cohort identified 12 CpG sites associated with type 2 diabetes. The association between these CpG sites and metabolic traits was measured in a further 1018 individuals [7]. MR analyses revealed that there was a likely causal effect of fasting glucose on cg00574958 (*CPT1A*). There was no evidence to suggest a causal effect of type 2 diabetes on methylation in this study [7].

In a study using genotype as a causal anchor to assess future risk of type 2 diabetes, DNAm did not appear to be on the causal pathway between known type 2 diabetes genetic risk variants and type 2 diabetes, with the exception of *KCNQ1* [16]. A further study of type 2 diabetes genetic risk variants that were also *cis-*mQTLs (defined as local variants <1 Mb from the DNAm site) assessed the causal pathway from methylation to type 2 diabetes using MR. This study identified CpG sites at four loci (*HNF1B*, *KCNJ11*, *IGF2BP2* and *WFS1*) that were likely to be causally associated with future risk of type 2 diabetes [17].

While efforts have been made to estimate the causal direction of effect between DNAm and type 2 diabetes, conclusions remain sparse and inconsistent. In part, this is because previous studies have used modestly sized datasets (<1000 individuals). The first aim of this study was to comprehensively investigate the causal direction of effect between DNAm and type 2 diabetes using a bidirectional two-sample MR (2SMR) approach, selecting genetic instruments that were (1) strongly associated with the exposure; (2) associated with the outcome solely via the exposure; and (3) not related to the confounders [18]. DNAm was assessed at CpG sites identified in the largest meta-EWAS of prevalent type 2 diabetes available in European populations (340 individuals with type 2 diabetes and 3088 control participants) [6]. The second aim of this study was to determine the functional role of CpGs stratified by their likely causal direction of effect on type 2 diabetes. We hypothesised that CpGs may have different causal directions of association with type 2 diabetes and that this may indicate their specific mechanism of action in type 2 diabetes onset and progression.

## Methods

### Study samples

#### Forward 2SMR: type 2 diabetes as causal of differences in DNAm at candidate CpGs

We extracted 148 genetic variants (SNPs) associated with type 2 diabetes from four DIAGRAM consortium GWAS [19] (see electronic supplementary material [ESM] Table 1). Three included multiethnic samples [20–22] and one included people of European ancestry [23]. We selected a SNP if it was (1) identified as an index variant in a GWAS meta-analysis; (2) found within a 99% credible set from an index variant, with an equal or higher posterior probability than the index variant; and (3) identified in the exome mapping close to a well-established locus for type 2 diabetes, with a minor allele frequency (MAF) >0.05. Two levels of significance were considered in the selection of SNPs: a genome-wide significance threshold of *p*<5.0×10^–8^ and a locus-wide significance threshold of *p*<1.0×10^–5^ (genome-wide complex trait analysis joint regression model). SNPs were excluded if they had incomplete risk allele data, no reported effect estimate (OR), no *p* value or a MAF <0.05. After quality control in MR-Base [24], the initial list of 148 type 2 diabetes SNPs was reduced to 62 SNPs (linkage disequilibrium [LD] *r*^2^<0.2) [25] in our outcome sample (ALSPAC-ARIES) (ESM Table 2).

**Fig. 1:**
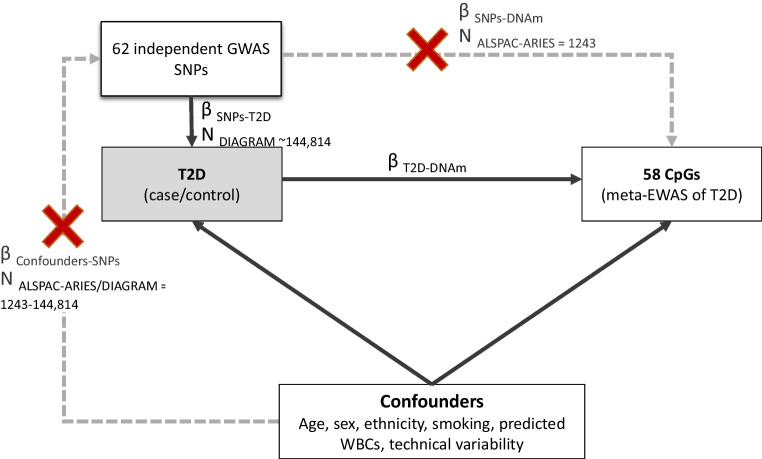
Study design of the forward 2SMR analysis investigating the causal effect of type 2 diabetes on differences in DNAm at 58 CpG sites. CpGs were previously identified in association with type 2 diabetes in a meta-EWAS. T2D, type 2 diabetes; WBC, white-blood cell

We used data from ALSPAC-ARIES [26–28] (ESM Methods S1) to extract estimates of the association between the 62 type 2 diabetes SNPs and DNAm levels at 58 CpG sites previously associated with prevalent type 2 diabetes at *p*<1.0×10^–5^ (six of 58 CpGs had an epigenome-wide *p*<1.33×10^–7^) [6]. For this analysis, we included cross-sectional data from 1243 middle-aged participants (age range 31–75 years) irrespective of their type 2 diabetes status. We could not use the Genetics of DNA Methylation Consortium (GoDMC) [29] as our outcome sample because type 2 diabetes SNPs, or related SNPs in high LD (*r*^2^>0.6), were associated with CpGs of interest at *p*>10^–8^, which was the maximum *p* value threshold used by GoDMC to report summary data. Figure 1 shows the study design used to conduct the forward 2SMR analysis.

#### Reverse 2SMR: DNAm at candidate CpGs as causal risk factors for type 2 diabetes

Using GoDMC summary data [29], we retrieved 41 mQTL associated with DNAm in blood at 31 of the 58 CpG sites previously reported in a meta-EWAS of type 2 diabetes [6] (ESM Methods S2). We selected mQTL with a *p*<10^–8^ for *cis*-mQTL (SNP <1 Mb from CpG site) and a *p*<10^–14^ for *trans*-mQTL (defined as distal variants >1 Mb from the CpG site). In total, five of 41 mQTL identified were *trans*-mQTL. When there were multiple mQTL for a single CpG, we used the *clump_data* function in the R package TwoSampleMR to select independent SNPs (ESM Methods S3). Data for each mQTL were obtained using a fixed-effect meta-analysis, with effect estimates interpreted as a unit change in inverse normal transformed residuals of DNAm, per additional effect allele.

We verified that mQTL used in the reverse MR were independent of SNPs for type 2 diabetes in the forward MR using a LD threshold of *r*^2^<0.01 to avoid bias in the causal estimate due to pleiotropic effects or reverse causation [30]. The correlation between type 2 diabetes SNPs and mQTL was calculated using Ldlink (version 3, https://ldlink.nci.nih.gov/) [31], selecting as the reference panel genetic data from European samples in the 1000 Genomes project phase 3.

**Fig. 2:**
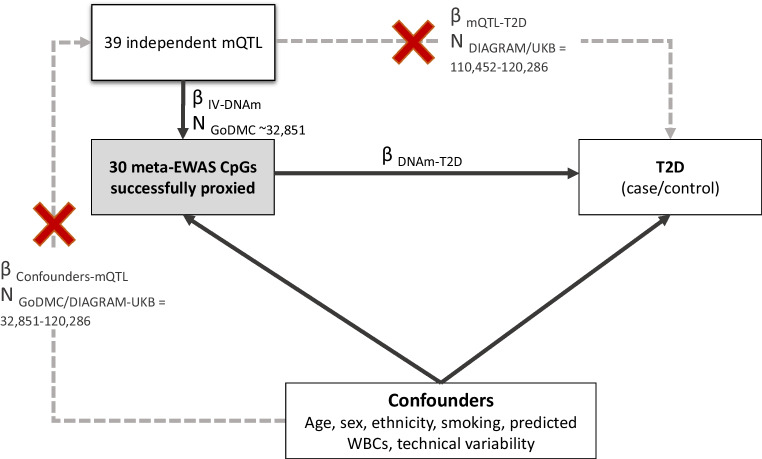
Study design of the reverse 2SMR analysis investigating the causal effect of differences in DNAm on type 2 diabetes risk. A total of 58 CpGs were previously identified in association with type 2 diabetes in a meta-EWAS, but only 30 of them were proxied by an mQTL SNP in GoDMC with available GWAS data for type 2 diabetes in MR-Base. IV, instrumental variable; T2D, type 2 diabetes; UKB, UK Biobank; WBC, white-blood cell

As our outcome sample, we selected the two largest type 2 diabetes GWAS available [20, 32]. Associations of mQTL with type 2 diabetes were extracted using the MRInstruments and TwoSampleMR R packages. When mQTL information was not available in the outcome sample, MR-Base looked for alternative SNPs with a LD *r*^2^>0.8 with the target mQTL SNP. After MR-Base data harmonisation, we were able to successfully extract outcome data for 39 of the initial 41 mQTL SNPs typing 30 meta-EWAS CpGs. The study design used to conduct the reverse 2SMR analysis is shown in Fig. 2.

### Statistical analyses

#### Forward 2SMR: conducting a SNP–CpG analysis in ALSPAC-ARIES

We measured associations between 62 independent type 2 diabetes SNPs and 58 CpG sites previously reported in a meta-EWAS of type 2 diabetes using a standardised protocol [29]. We conducted linear regressions with the genotype for type 2 diabetes as the exposure and DNAm (inverse normal transformed residuals) at each CpG site as the outcome using an additive genetic model. Briefly, in the first stage of the analysis, we extracted complete genetic and DNAm data for 1243 middle-adults in ALSPAC-ARIES (age range 31–75 years) and performed quality control on each dataset separately (ESM Methods S4–S6). For genome-wide SNPs and CpG sites that passed the quality control, we selected only those corresponding to type 2 diabetes SNPs and meta-EWAS CpGs, respectively, and conducted regression analyses using the MatrixeQTL R package version 2.3 [33]. We tested the direct effect of the genotype for type 2 diabetes on DNAm at meta-EWAS CpGs at *p*<1.4×10^–5^ or α=0.05/62 type 2 diabetes SNPs×58 CpGs. We interpreted effect estimates in the SNP–CpG analysis as the difference in residuals of DNAm (inverse normal transformed values), per additional risk allele for type 2 diabetes.

Mendelian randomisation and MR-Base analysis MR is a statistical method used to infer causality in observational associations that has been documented in detail elsewhere [15]. For this study, we carried out a 2SMR analysis using summary data from two independent but comparable populations [34], both with moderate power. MR-Base was used to conduct causal analyses [24]. Further detail is provided in ESM Methods S3. Estimated causal effects were interpreted as the effect of type 2 diabetes on a unit increase in residuals of DNAm (forward 2SMR) or as the odds of type 2 diabetes per unit increase in residuals of DNAm (reverse 2SMR).

#### Determining the true direction of association

For associations analysed bidirectionally, we inferred the likely causal direction using the causal effect estimate with the smallest *p* value, which was also consistent with the direction of association found in the observational analysis (i.e. meta-EWAS of type 2 diabetes). ‘Inconclusive’ associations with bidirectional MR data had a *p* value >0.1 in each direction of the analysis. Associations with analysed MR data that were in a single direction only were regarded as ‘inconclusive, single direction’.

We used the Steiger test to confirm that the true direction of association was the one specified in the analysis. This test has its limitations [35] and it may perform better when using continuous rather than categorical exposures (i.e. more reliability when testing directionality in the reverse rather than in the forward 2SMR analysis).

#### Functional inspection of MR signals using publicly available databases

We classified meta-EWAS CpGs into three subgroups based on their most likely causal direction of association with type 2 diabetes: (1) type 2 diabetes causal of DNAm variation; (2) DNAm causal of type 2 diabetes risk; and (3) inconclusive direction of association. We looked for *cis*-eQTM associated with meta-EWAS CpGs in the BIOS QTL browser (https://molgenis26.gcc.rug.nl/downloads/biosqtlbrowser/) [36]. In addition, results from the EWAS Catalog [37] were grouped into related phenotypes [38] and tested for enrichment among the 58 meta-EWAS CpGs analysed. Enrichment of each CpG subgroup for specific phenotypes was reported using ORs and 95% CIs. The *p *values in the enrichment analysis were one-sided. We considered evidence of enrichment at *p*<0.05 per traits analysed.

### Ethical disclosure

Ethical approval for ALSPAC and its substudy ARIES was obtained from the ALSPAC Ethics and Law Committee and the Local Research Ethics Committees. Consent for use of biological samples was collected in accordance with the Human Tissue Act (2004).

## Results

### Forward 2SMR: type 2 diabetes is suggestively associated with lower DNAm at cg20812370 (*PBX1*), previously identified in a meta-EWAS of type 2 diabetes

Summary statistics of type 2 diabetes–SNP associations are shown in ESM Table 2. Association estimates between type 2 diabetes SNPs and CpGs are presented in ESM Table 3.

**Fig. 3:**
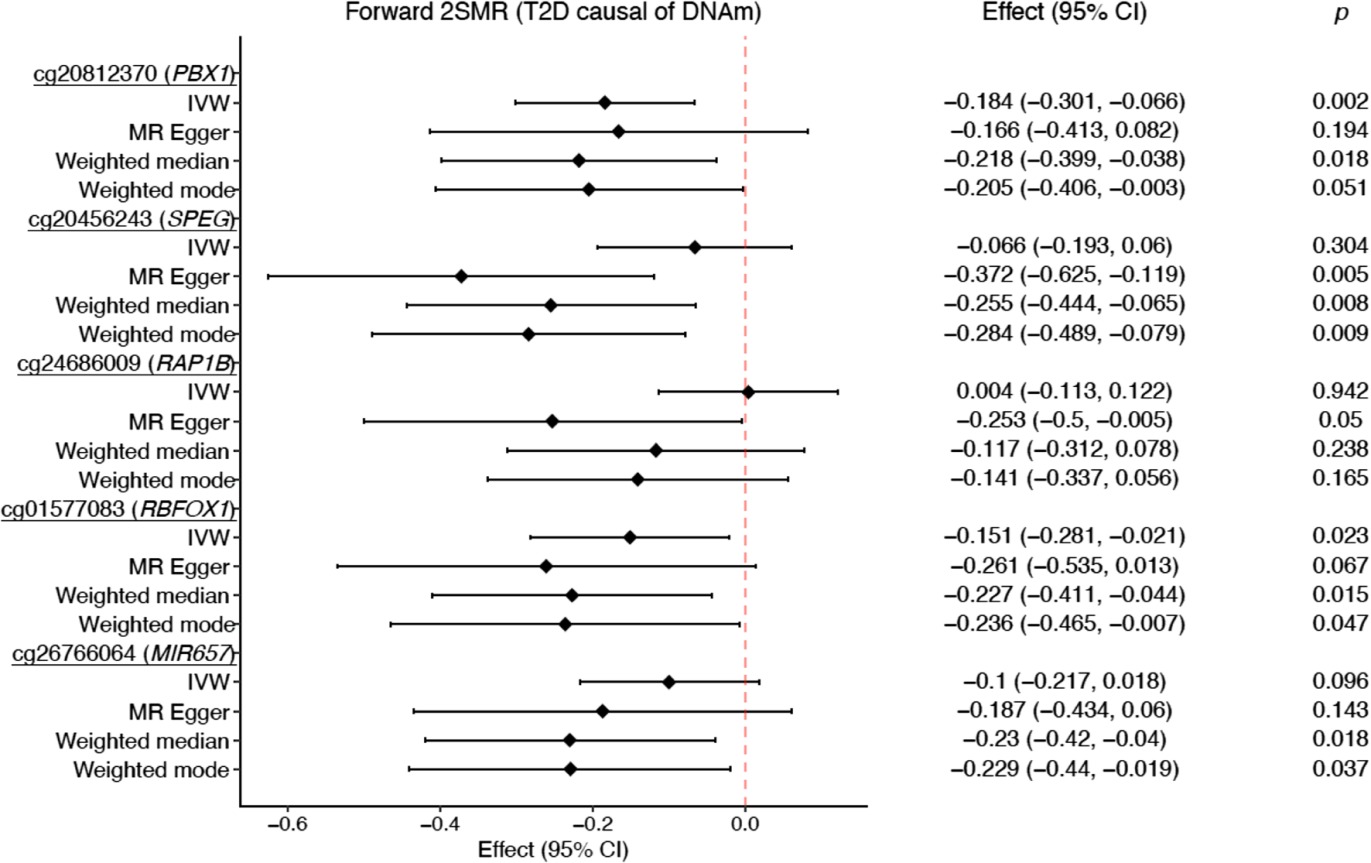
Forest plot showing causal effect estimates from the 2SMR analysis of the effect of prevalent type 2 diabetes on the difference in DNAm at five meta-EWAS CpGs with the strongest evidence of causality in the forward 2SMR analysis. The black diamond represents the mean causal effect for each CpG and MR method and the horizontal line shows the 95% CI. *p*, unadjusted *p* value. Associations were borderline significant at an unadjusted *p*<0.05 and significant at an adjusted *p*<0.001. T2D, type 2 diabetes

Using forward 2SMR, we observed evidence of causality (adjusted *p*<0.001 or unadjusted *p*<0.05) between type 2 diabetes and lower levels of DNAm at the CpGs cg20812370 (*PBX1*) (*p*=0.002) and cg01577083 (*RBFOX1*) (*p*=0.023) (Fig. 3, ESM Figs 1 and 2, ESM Methods S7). The causal effects at both CpGs were directionally consistent with the results of the observational analysis (meta-EWAS), but absolute effect sizes were always larger in the MR analysis than in the observational analysis (Table 1). For associations at cg20812370 (*PBX1*) and cg01577083 (*RBFOX1*), there was little evidence of heterogeneity in the effect of 62 type 2 diabetes SNPs on DNAm levels based on the results of the Cochran’s *Q* test (*Q* range 52.9–74.8, *p* value range 0.10–0.75).

**Table 1:** Observational associations and estimates of causal effect between prevalent type 2 diabetes and differences in DNAm at five CpGs observed to have the strongest evidence of causality in the forward 2SMR analysis

CpG	Chr	Nearest gene	Meta-EWAS of type 2 diabetes^a^			MR IVW regression	
			Effect (95% CI)	*p* value	*n*	Effect (95% CI)	*p* value
cg20812370	1	*PBX1*	−0.007 (−0.009, −0.004)	7.40×10^−7^	3428	−0.184 (−0.301, −0.066)	0.002
cg20456243	2	*SPEG*	−0.007 (−0.011, −0.004)	9.99×10^−6^	3428	−0.066 (−0.193, 0.06)	0.304
cg24686009	12	*RAP1B*	−0.002 (−0.003, −0.001)	1.19×10^−6^	3428	0.004 (−0.113, 0.122)	0.942
cg01577083	16	*RBFOX1*	−0.011 (−0.016, −0.006)	7.93×10^−6^	3428	−0.151 (−0.281, −0.021)	0.023
cg26766064	17	*MIR657*	−0.007 (−0.009, −0.004)	5.17×10^−6^	3428	−0.100 (−0.217, 0.018)	0.096

^a^Meta-EWAS of prevalent type 2 diabetes (*n*=3428) [6]

Associations were considered significant at *p*<0.001 or α=0.05/58 CpGs analysed in the forward 2SMR analysis

Using MR-Egger, weighted mode and weighted median as sensitivity analyses, we observed four CpGs with evidence of causality with type 2 diabetes at *p*<0.05: the previously detected cg20812370 (*PBX1*) and cg01577083 (*RBFOX1*) plus cg20456243 (*SPEG*) and cg26766064 (*MIR657*) (Table 2). In all cases, the magnitude and direction of the effect estimates were similar across analyses, but smaller *p* values were generally seen when using the weighted median than when using the weighted mode and MR-Egger regressions. The results of these sensitivity analyses were directionally consistent with estimates of the inverse variance weighted (IVW) regression and with the observational analysis (meta-EWAS).

**Table 2:** Estimates of the causal effect between prevalent type 2 diabetes and difference in DNAm at five CpGs using additional MR sensitivity analyses for the forward 2SMR analysis

CpG	Chr	Nearest gene	MR-Egger regression		Weighted median		Weighted mode	
			Effect (95% CI)	*p* value	Effect (95% CI)	*p* value	Effect (95% CI)	*p* value
cg20812370	1	*PBX1*	−0.166 (−0.413, 0.082)	0.194	−0.218 (−0.399, −0.038)	0.018	−0.205 (−0.406, −0.003)	0.051
cg20456243	2	*SPEG*	−0.372 (−0.625, −0.119)	0.005	−0.255 (−0.444, −0.065)	0.008	−0.284 (−0.489, −0.079)	0.009
cg24686009	12	*RAP1B*	−0.253 (−0.5, −0.005)	0.050	−0.117 (−0.312, 0.078)	0.238	−0.141 (−0.337, 0.056)	0.165
cg01577083	16	*RBFOX1*	−0.261 (−0.535, 0.013)	0.067	−0.227 (−0.411, −0.044)	0.015	−0.236 (−0.465, −0.007)	0.047
cg26766064	17	*MIR657*	−0.187 (−0.434, 0.06)	0.143	−0.230 (−0.420, −0.040)	0.018	−0.229 (−0.44, −0.019)	0.037

MR-Egger: sensitivity analysis to account for horizontal pleiotropy. Weighted median and weighted mode: sensitivity analyses that allow for some instruments to be invalid, while generating unbiased causal estimates with the set of valid proxies

Associations were considered significant at *p*<0.001 or α=0.05/58 CpGs analysed in the forward 2SMR analysis

Overall, in the forward 2SMR analysis, we found no evidence of weak instrument bias based on values of the *F* statistic, which ranged from 19.7 to 274.8 (strong instrument if *F* statistic >10).

### Reverse 2SMR: elevated DNAm at cg25536676 (*DHCR24*) is associated with an increased risk of type 2 diabetes

Summary estimates of the association between the 39 mQTL SNPs and 30 meta-EWAS CpGs are shown in ESM Table 4. Association estimates of the 39 mQTL SNPs with type 2 diabetes are presented in ESM Table 5. Overall, none of these mQTL was directly associated with type 2 diabetes at the GWAS significance threshold (*p*<5.0×10^–8^). Four mQTL tagging the CpGs cg08857797 (*VPS25*), cg00144180 (*HDAC4*), cg16765088 (*SYNM*) and cg25536676 (*DHCR24*) were suggestively associated with type 2 diabetes, with an unadjusted GWAS *p*<0.05 (ESM Table 5).

In the reverse 2SMR analysis, we identified a strong causal effect (*p*<0.002 or α=0.05/30 CpGs) between cg25536676 (*DHCR24*) and type 2 diabetes using the Wald ratio (Table 3). For this CpG, we found an opposite direction of association between the causal and the observational analysis. Similar causal associations with type 2 diabetes were seen at cg25536676 using the IVW regression (ESM Table 6), with no evidence of heterogeneity (Cochran’s *Q*=2.4, *p*=0.30). The Steiger test suggested that the true direction of the association at cg25536676 was from DNAm to type 2 diabetes (Steiger *p*=3.8×10^–147^, *R*^2^ for CpG=0.04, *R*^2^ for type 2 diabetes=5.3×10^–5^). We conducted MR sensitivity analysis for the association at cg25536676 (ESM Table 6), but these results may be unreliable because of the small number of mQTL used as instruments (*n*=3).

**Table 3:** Observational associations and estimates of causal effect between prevalent type 2 diabetes and differences in DNAm at five CpGs observed to have the strongest evidence of causality in the reverse 2SMR analysis

CpG	Chr	Nearest gene	GWAS^a^	Meta-EWAS of type 2 diabetes^b^		MR Wald ratio		
				Effect (95% CI)	*p* value	OR (95% CI)	Effect (95% CI)	*p* value
cg25536676	1	*DHCR24*	Mahajan et al [20]	−0.008 (−0.011, −0.004)	5.39×10^−6^	1.43 (1.15, 1.78)	0.36 (0.14, 0.58)	0.001
cg11851382	1	*PPAP2B* (*PLPP3*)	Wood et al [32]	−0.008 (−0.011, −0.005)	6.42×10^−6^	0.29 (0.12, 0.67)	−1.24 (−2.09, −0.4)	0.004
cg10082515	7	*EIF3IP1*	Wood et al [32]	−0.013 (−0.019, −0.008)	7.46×10^−6^	0.68 (0.52, 0.89)	−0.38 (−0.65, −0.11)	0.005
cg07212837	8	*GRINA*	Mahajan et al [20]	0.006 (0.004, 0.009)	3.28×10^−6^	1.09 (1.02, 1.17)	0.09 (0.02, 0.16)	0.018
cg16765088	15	*SYNM*	Wood et al [32]	−0.011 (−0.014, −0.007)	5.50×10^−10^	1.72 (1.02, 2.9)	0.54 (0.02, 1.07)	0.040

^a^GWAS used to extract information on the genotype–outcome associations

^b^Meta-EWAS of prevalent type 2 diabetes (*n*=3428) [6]

Associations were considered significant at *p*<0.002 or α=0.05/30 CpGs analysed in the reverse 2SMR analysis

We observed evidence (unadjusted *p*<0.05) of a causal effect of DNAm on type 2 diabetes at four other CpG sites (Table 3). Estimates of the causal and the observational analysis were consistent for most of these associations, except for the CpG in *SYNM*. No other MR analyses were carried out at these four CpGs as they were proxied by a single mQTL.

**Fig. 4:**
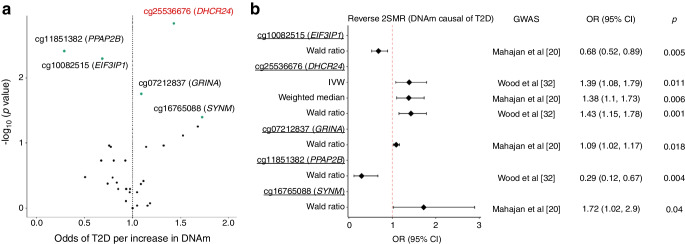
Summary of evidence from the reverse 2SMR analysis for the association between inverse normal transformed residuals of DNAm at five meta-EWAS CpGs and risk of prevalent type 2 diabetes. The CpGs illustrated showed the smallest *p* values in the reverse 2SMR analysis. (**a**) Volcano plot showing MR estimates using the Wald ratio and a single SNP as a proxy for each CpG analysed. The CpG highlighted in red (cg25536676 [*DHCR24*]) was identified as causally associated with type 2 diabetes (*p*=0.001). The green dots represent CpGs with an unadjusted *p*<0.05 or –log_10_ (*p* value) >1.3 in the 2SMR analysis. The vertical dashed line represents the line of null associations at OR=1.0. (**b**) Forest plot showing means and SEMs of the causal estimates of the association between DNAm and T2D (outcome) for the top five CpGs identified in the reverse 2SMR analysis. The results for each CpG are shown according to the MR method applied, which differed only for the CpG cg25536676 (*DHCR24*), proxied by three mQTL. T2D, type 2 diabetes

Overall, the results of the Steiger test confirmed that, for the top CpGs identified in the reverse 2SMR analysis, the true direction of association was from DNAm to type 2 diabetes (Steiger *p* value range 1.2×10^–164^–1.5×10^–94^, mean *R*^2^ for CpGs=0.03 vs mean *R*^2^ for type 2 diabetes=2.3×10^–5^). A mean value of the *F* statistic of 20.3 suggested a low probability of obtaining biased results as a result of weak instruments. Figure 4 provides volcano and forest plots summarising the results of the reverse 2SMR analysis.

### Bidirectional interrogation of associations identified in the 2SMR analysis

To determine the true direction of the associations with type 2 diabetes, we summarised the bidirectional causal effects derived from the 2SMR analysis. For the seven top CpGs with bidirectional 2SMR data (two detected in the forward 2SMR and five in the reverse 2SMR analysis) (Table 4), we confirmed that the true direction of association was the one we originally interrogated in the single-direction analysis. At cg10082515 (*EIF3IP1*), we observed the same direction of association between the observational and the bidirectional causal analysis (Table 4). For four of the top six CpGs previously identified in a meta-EWAS of type 2 diabetes that had bidirectional 2SMR data (CpGs in *TXNIP*, *HDAC4*, *SYNM* and *ABCG1*), the observational and causal effects were consistent only at *HDAC4* (cg00144180)*.* At this CpG, the MR results suggested that the true direction of association was from DNAm to type 2 diabetes, similar to what we observed at the CpG cg16765088 in *SYNM* (ESM Table 7). For the CpGs cg19693031 (*TXNIP*) and cg06500161 (*ABCG1*), the results of the 2SMR analysis were inconclusive (i.e. large *p* values in both directions of the analysis). The CpG cg00574958 in *CPT1A*, previously shown as causally associated with fasting glucose [7], was not associated with type 2 diabetes in the single direction in which it was analysed (forward 2SMR: type 2 diabetes to DNAm).

**Table 4:** Bidirectional comparison of MR estimates obtained for CpGs associated with type 2 diabetes with significance or borderline significance in either direction of the 2SMR analysis

CpG	Nearest gene	Meta-EWAS of T2D^a^		Forward 2SMR		Reverse 2SMR		Likely causal direction
				(T2D → DNAm)		(DNAm → T2D)		
		Estimate (95% CI)	*p* value	Estimate (95% CI)	*p* value (IVW)	Estimate (95% CI)	*p* value (Wald ratio)	
cg11851382	*PPAP2B*^b^ (*PLPP3*)	−0.008 (−0.011, −0.004)	6.42×10^−6^	0.03 (−0.09, 0.15)	0.603	−1.24 (−2.09, −0.40)	0.004	DNAm → T2D
cg20812370	*PBX1*^c^	−0.007 (−0.009, −0.004)	7.40×10^−7^	−0.18 (−0.30, −0.07)	0.002	–	–	T2D → DNAm
cg25536676	*DHCR24*^b^	−0.008 (−0.011, −0.004)	5.39×10^−6^	0.04 (−0.08, 0.16)	0.490	0.36 (0.14, 0.58)	0.001	DNAm → T2D
cg20456243	*SPEG*^c^	−0.007 (−0.011, −0.004)	9.99×10^−6^	−0.07 (−0.19, 0.06)	0.304	0.08 (−0.12, 0.29)	0.429	T2D → DNAm
cg10082515	*EIF3IP1*^b^	−0.013 (−0.019, −0.008)	7.46×10^−6^	−0.05 (−0.16, 0.07)	0.443	−0.38 (−0.65, −0.11)	0.005	DNAm → T2D
cg07212837	*GRINA*^b^	0.006 (0.004, 0.009)	3.28×10^−6^	−0.07 (−0.2, 0.06)	0.304	0.09 (0.02, 0.16)	0.018	DNAm → T2D
cg24686009	*RAP1B*^d^	−0.002 (−0.003, −0.001)	1.19×10^−6^	0.004 (−0.11, 0.12)	0.942	–	–	Inconclusive
cg16765088	*SYNM*^b^	−0.011 (−0.014, −0.007)	5.50×10^−10^	−0.08 (−0.20, 0.04)	0.188	0.54 (0.02, 1.07)	0.04	DNAm → T2D
cg01577083	*RBFOX1*^c^	−0.011 (−0.016, −0.006)	7.93×10^−6^	−0.15 (−0.28, −0.02)	0.023	0.00 (−0.32, 0.32)	1.00	T2D → DNAm
cg26766064	*MIR657*^c^	−0.007 (−0.009, −0.004)	5.17×10^−6^	−0.10 (−0.22, 0.02)	0.096	–	–	T2D → DNAm

^a^Meta-EWAS of prevalent type 2 diabetes (*n*=3428) [6]

Associations were considered significant at *p*<1.33×10^–7^ in the meta-EWAS of T2D, at *p*<0.001 in the forward 2SMR analysis and at *p*<0.002 in the reverse 2SMR analysis

^b^CpGs identified as having significance or borderline significance (*p*<0.05) in the reverse 2SMR analysis. CpGs did not have significance or borderline significance in the forward 2SMR analyses

^c^CpGs identified as having borderline significance (*p*<0.05) in the forward 2SMR analysis, either in IVW (this table) or in sensitivity analyses (MR-Egger, weighted mode or weighted median; see Table 2 for test statistics)

^d^The causal direction for CpGs was deemed to be inconclusive when 2SMR data were available in only one direction and *p*>0.1, or if 2SMR data were available bidirectionally but in both cases *p*>0.1

### In silico interrogation of the functional role of meta-EWAS CpGs analysed by bidirectional 2SMR

Results of the in silico analysis suggested that CpGs included in the bidirectional 2SMR analysis were enriched for eQTMs (OR 5.4, 95% CI 2.7, 11.1, *p*=1.5×10^–7^) (Table 5). We also interrogated the enrichment of meta-EWAS CpGs for traits included in the EWAS Catalog, categorising CpGs into three groups according to their likely causal direction of effect based on the bidirectional 2SMR analysis (see Methods, Functional inspection of MR signals using publicly available databases). Common to all groups was enrichment for ancestry/ethnicity and anthropometric and cardiometabolic traits. Unique to the first group (type 2 diabetes likely to be causal of DNAm) was enrichment for neurological and perinatal traits, while unique to the second group (DNAm likely to be causal of type 2 diabetes) was enrichment for lipid lipoproteins, alcohol and metabolites. In the third group of CpGs with an inconclusive direction of association, we found enrichment for all the above traits in addition to age and tissue type. Forest plots summarising the results of the enrichment analysis are presented in ESM Fig. 3.

**Table 5:** Look up of eQTMs among meta-EWAS CpGs included in the bidirectional 2SMR analysis

CpG	Transcript	Chr	CpG position	Transcript position	Nearest gene	Beta (SE)^a^	*p* value^a^	FDR
cg14476101	ENSG00000092621	1	120255944	120202421	*PHGDH*	0.34 (0.04)	2.1×10^−55^	<0.001
cg19693031	ENSG00000117289	1	145441552	145438469	*TXNIP*	−0.12 (0.04)	7.1×10^−8^	9.3×10^−6^
cg12593793	ENSG00000160789	1	156074135	156052364	*LMNA*	−0.21 (0.04)	5.1×10^−18^	<0.001
cg20456243	ENSG00000072195	2	220352428	220299568	*SPEG*	0.08 (0.04)	2.4×10^−5^	6.0×10^−3^
cg00574958	ENSG00000110090	11	68607622	68611878	*CPT1A*	−0.17 (0.04)	3.1×10^−20^	<0.001
cg11024682	ENSG00000072310	17	17730046	17740325	*SREBF1*	−0.19 (0.04)	4.5×10^−15^	<0.001
cg18181703	ENSG00000184557	17	76354573	76356158	*SOCS3*	0.13 (0.04)	1.1×10^−6^	3.3×10^−4^
cg27037013	ENSG00000237945	21	35320619	35287838	*LINC00649*	0.14 (0.04)	4.8×10^−7^	1.3×10^−4^
cg06500161	ENSG00000160179	21	43656587	43619799	*ABCG1*	−0.32 (0.04)	2.2×10^−37^	<0.001

^a^Association estimates as retrieved from the BIOS QTL browser [36]

## Discussion

In this study we conducted a bidirectional 2SMR analysis to investigate the direction of causality between prevalent type 2 diabetes and DNAm at 58 CpGs previously identified in a meta-EWAS of type 2 diabetes among European populations. In the forward 2SMR analysis, in which we examined if type 2 diabetes was causal of differences in DNAm, we interrogated causality at all 58 meta-EWAS CpGs. No tests passed our *p* value threshold. The CpG cg20812370 (*PBX1*) had the smallest *p* value (*p*=0.002), with similar results found in sensitivity analyses. For the reverse 2SMR analysis, in which DNAm was the exposure, we tested causality at 30 of the 58 meta-EWAS CpGs. We demonstrated that elevated DNAm at cg25536676 (*DHCR24*) was causally associated with an increased risk of type 2 diabetes, which is inconsistent with previous observational evidence. By assessing the MR and observational estimates, we attempted to infer the likely causal direction for the 30 CpGs analysed bidirectionally. Considering the consistency of the direction of causality with estimates from the observational analysis, as well as the smallest *p* value in the 2SMR analysis (*p*<0.1), we concluded that, for 15 (50%) of the 30 CpGs, DNAm was likely to be causal of type 2 diabetes and for 10 (33%) of the 30 CpGs type 2 diabetes was likely to be causal of DNAm; for the remaining five (17%) CpGs the results were inconclusive. In silico analyses showed that some of the CpGs analysed correspond to eQTMs, and that CpGs may be enriched for specific traits depending on the direction of causality predicted by the 2SMR analysis.

### Findings of the forward 2SMR analysis

No associations were identified in the forward 2SMR analysis. This may be because there were power limitations of the data available for the analysis of the genotype–outcome association (i.e. ALSPAC-ARIES vs GoDMC). We could not confirm the previously reported nominal and robust associations of *CPT1A* methylation with type 2 diabetes [11] and fasting glucose [7] in our 2SMR analysis.

### Findings of the reverse 2SMR analysis

The results of the bidirectional 2SMR analysis suggested that most associations identified relate to differences in DNAm influencing the risk of type 2 diabetes, rather than the opposite, with the most robust evidence obtained at cg25536676 (*DHCR24*). We found that increased levels of inverse normal transformed residuals of DNAm at this CpG were associated with a 43% higher risk of type 2 diabetes and this result was consistent in various sensitivity analyses; however, this was opposite to the result found in the observational analysis [6]. One reason for the opposite direction of effect observed between the observational meta-EWAS and the MR analysis at cg25536676 is residual confounding of the observational analysis. Potential confounding factors include BMI, lipid profile or medication status [39]. Observational analysis at other *DHCR24* CpG sites has also identified an inverse relationship between methylation and incident type 2 diabetes but this was attenuated after adjusting for smoking, BMI and follow-up time [12]. We are not aware of other studies assessing the causal association between a CpG in *DHCR24* and type 2 diabetes, but there is evidence of causality with LDL-cholesterol (LDL-C) levels in the same locus [40] and the direction of association is consistent between both traits (type 2 diabetes and LDL-C). Thus, the association we found in the MR analysis between type 2 diabetes and DNAm at cg25536676 (*DHCR24*) may reflect an underlying effect of LDL-C on DNAm at *DHCR24*. No evidence of heterogeneity or pleiotropic effects was found for the association at cg25536676 (*DHCR24*). DNAm at cg25536676 has been previously found in association with total cholesterol [41] in whole blood and with perinatal traits such as gestational age [42] and birthweight [43] in cord blood.

Other CpGs in the region of *DHCR24* (cg17901584 and cg27168858) have been associated with HDL-cholesterol (HDL-C) [41], triglycerides [44], fasting insulin [45, 46], BMI, HbA_1c_, incident type 2 diabetes [46], statin use [39], waist circumference [47] and LDL-C [40]. Both nearby CpGs in *DHCR24* were positively associated with levels of HDL-C and LDL-C, but only cg17901584 was negatively associated with the remaining cardiometabolic traits. Using causal analysis to overcome the effect of residual confounding, it was previously demonstrated that LDL-C, but not HDL-C, was causally associated with methylation at *DHCR24* [40]. In addition, it was suggested that the causal direction of effect was from LDL-C to changes in DNAm at *DHCR24* and not the opposite. Our MR result at the type 2 diabetes-related CpG cg25536676 is directionally consistent with the association identified with LDL-C at the nearby CpG in *DHCR24*. Evidence of causality has been identified between BMI (exposure) and *DHCR24* DNAm (outcome) [46]. Because obesity and lipid dysregulation are well-known hallmarks of type 2 diabetes, and because *DHCR24* encodes for an enzyme related to the metabolism of cholesterol (3-hydroxysterol-24 reductase) [41], an association between methylation at *DHCR24* and type 2 diabetes is plausible. Thus far, evidence suggests that cg25536676 (*DHCR24*) may act as a causal mediator in the association between known risk factors (i.e. LDL-C) and type 2 diabetes. However, a formal two-step 2SMR analysis needs to be implemented to prove this hypothesis. For LDL-C, there is observational [48] and causal [49] evidence supporting an inverse association between circulating LDL-C levels and type 2 diabetes. This association is in the opposite direction to that proposed for a putative mediating role of cg25536676 (*DHCR24*) in LDL-C metabolism and type 2 diabetes. Taken together, our results suggest that cg25536676 may be a new DNAm target for the early detection and treatment of type 2 diabetes.

### Functional analysis

The in silico analysis using data from the previous EWAS showed that CpGs detected as being potentially causal of type 2 diabetes may be associated with lipid lipoproteins, metabolites and anthropometric and cardiometabolic traits, all of which are involved in the onset of type 2 diabetes. For comparison, CpGs that were likely to be secondary to the effects of type 2 diabetes (in *PBX1, SPEG, MIR657* and *RBFOX1*) based on the results of the 2SMR analysis were enriched for perinatal and neurological traits in addition to cardiometabolic and anthropometric traits. Although not conclusive, these results indicate that DNAm may be involved in the development and progression of type 2 diabetes through different mechanisms related to specific stages in the pathophysiology of the disease. We hypothesise that the observed enrichment for ancestry/ethnicity among type 2 diabetes CpG sites identified in European populations may highlight common confounders or exposures (e.g. differences in cell composition or type 2 diabetes risk factors) between populations [50].

### Strengths and limitations

Our study has several strengths. First, we leveraged large existing datasets to extract genetic associations with type 2 diabetes and DNAm. Second, we analysed causality bidirectionally to disentangle the true direction of association and ruled out reverse causation at CpGs previously detected in the context of prevalent type 2 diabetes. We also conducted sensitivity analyses to validate assumptions of the MR analysis and we demonstrated that in each direction of the analysis we were unlikely to be affected by weak instrument bias. In addition, we ran in silico functional analyses to facilitate biological interpretation of the MR findings. Study limitations included the use of a smaller outcome sample in the forward 2SMR analysis. In the reverse 2SMR analysis (DNAm to type 2 diabetes), we had few proxies to predict DNAm at the CpGs of interest (~1 mQTL/CpG). This prevented us from obtaining stronger evidence of causality and conducting MR sensitivity analyses. Because up to 9.2% overlap was present between the exposure and the outcome samples in the reverse 2SMR analysis, the results need to be interpreted with caution because of potential bias. CpGs were selected from an observational EWAS of type 2 diabetes in European populations, which may limit the generalisability of the results to other population groups. Use of blood DNA rather than metabolically active tissue samples may limit interpretability. Finally, the validity of the observational and causal associations at *DHCR24* need to be confirmed before this evidence can be clinically implemented in the early detection and treatment of type 2 diabetes. Future studies would benefit from the use of multiethnic cohorts throughout the observational and causal inference stages of the analysis. Similarly, a two-step 2SMR analysis may be required to investigate the role of DNAm as a potentially causal mediator between known risk factors and type 2 diabetes.

### Conclusions

This study assessed causality between DNAm and type 2 diabetes in a bidirectional 2SMR framework at CpG sites identified from a meta-EWAS of prevalent disease. We had more power to identify causality from DNAm to type 2 diabetes than in the reverse direction, for which *PBX1* was the signal most likely to be associated with type 2 diabetes. We demonstrated that a CpG at *DHCR24*, a gene related to the metabolism of lipids, was causally associated with type 2 diabetes. Further validation of causal associations is needed using larger and better-powered samples to extract genetic associations, especially when DNAm is the exposure.

## Supplementary Information

Below is the link to the electronic supplementary material.

Supplementary file1 (PDF 1577 KB)

## Data Availability

ALSPAC data used for this submission will be made available on request to the ALSPAC executive committee (ALSPAC-exec@bristol.ac.uk). The ALSPAC data management plan (available at www.bristol.ac.uk/alspac/researchers/access/) describes in detail the policy regarding data sharing, which takes place through a system of managed open access.

## Abbreviations

2SMR: Two-sample Mendelian randomisation
ALSPAC: Avon Longitudinal Study of Parents and Children
ARIES: Accessible Resource for Integrated Epigenomic Studies
DNAm: DNA methylation
GoDMC: Genetics of DNA Methylation Consortium
GWAS: Genome-wide association study
HDL-C: HDL-cholesterol
LD: Linkage disequilibrium
LDL-C: LDL-cholesterol
MAF: Minor allele frequency
Meta-EWAS: Meta-analysis of epigenome-wide association studies
mQTL: Methylation quantitative trait loci
MR: Mendelian randomisation

## References

[1] Florath I, Butterbach K, Heiss J (2016). Type 2 diabetes and leucocyte DNA methylation: an epigenome-wide association study in over 1,500 older adults. Diabetologia.

[2] Kulkarni H, Kos MZ, Neary J (2015). Novel epigenetic determinants of type 2 diabetes in Mexican-American families. Hum Mol Genet.

[3] Soriano-Tárraga C, Jiménez-Conde J, Giralt-Steinhauer E (2016). Epigenome-wide association study identifies TXNIP gene associated with type 2 diabetes mellitus and sustained hyperglycemia. Hum Mol Genet.

[4] Al Muftah WA, Al-Shafai M, Zaghlool SB (2016). Epigenetic associations of type 2 diabetes and BMI in an Arab population. Clin Epigenetics.

[5] Meeks KAC, Henneman P, Venema A (2017). An epigenome-wide association study in whole blood of measures of adiposity among Ghanaians: the RODAM study. Clin Epigenetics.

[6] Juvinao-Quintero DL, Marioni RE, Ochoa-Rosales C (2021). DNA methylation of blood cells is associated with prevalent type 2 diabetes in a meta-analysis of four European cohorts. Clin Epigenetics.

[7] Kim H, Bae JH, Park KS, Sung J, Kwak SH (2021). DNA methylation changes associated with type 2 diabetes and diabetic kidney disease in an East Asian population. J Clin Endocrinol Metab.

[8] Albao DS, Cutiongco-de la Paz EM, Mercado ME (2019). Methylation changes in the peripheral blood of Filipinos with type 2 diabetes suggest spurious transcription initiation at TXNIP. Hum Mol Genet.

[9] Dayeh T, Tuomi T, Almgren P (2016). DNA methylation of loci within ABCG1 and PHOSPHO1 in blood DNA is associated with future type 2 diabetes risk. Epigenetics.

[10] Chambers JC, Loh M, Lehne B (2015). Epigenome-wide association of DNA methylation markers in peripheral blood from Indian Asians and Europeans with incident type 2 diabetes: a nested case-control study. Lancet Diabetes Endocrinol.

[11] Cardona A, Day FR, Perry JRB (2019). Epigenome-wide association study of incident type 2 diabetes in a British population: EPIC-Norfolk Study. Diabetes.

[12] Fraszczyk E, Spijkerman AMW, Zhang Y (2022). Epigenome-wide association study of incident type 2 diabetes: a meta-analysis of five prospective European cohorts. Diabetologia.

[13] Kim K, Joyce BT, Zheng Y (2021). DNA methylation GrimAge and incident diabetes: the Coronary Artery Risk Development in Young Adults (CARDIA) study. Diabetes.

[14] Wang Z, Peng H, Gao W (2021). Blood DNA methylation markers associated with type 2 diabetes, fasting glucose, and HbA1c levels: an epigenome-wide association study in 316 adult twin pairs. Genomics.

[15] Davey Smith G, Hemani G (2014). Mendelian randomization: genetic anchors for causal inference in epidemiological studies. Hum Mol Genet.

[16] Elliott HR, Shihab HA, Lockett GA (2017). Role of DNA methylation in type 2 diabetes etiology: using genotype as a causal anchor. Diabetes.

[17] Richardson TG, Haycock PC, Zheng J (2018). Systematic Mendelian randomization framework elucidates hundreds of CpG sites which may mediate the influence of genetic variants on disease. Hum Mol Genet.

[18] Davies NM, Holmes MV, Davey Smith G (2018). Reading Mendelian randomisation studies: a guide, glossary, and checklist for clinicians. BMJ.

[19] DIAGRAM. The DIAGRAM consortium. Available from: https://diagram-consortium.org. Accessed 25 July 2022

[20] Mahajan A, Go MJ, Zhang W (2014). Genome-wide trans-ancestry meta-analysis provides insight into the genetic architecture of type 2 diabetes susceptibility. Nat Genet.

[21] Morris AP, Voight BF, Teslovich TM (2012). Large-scale association analysis provides insights into the genetic architecture and pathophysiology of type 2 diabetes. Nat Genet.

[22] Fuchsberger C, Flannick J, Teslovich TM (2016). The genetic architecture of type 2 diabetes. Nature.

[23] Gaulton KJ, Ferreira T, Lee Y (2015). Genetic fine mapping and genomic annotation defines causal mechanisms at type 2 diabetes susceptibility loci. Nat Genet.

[24] Hemani G, Zheng J, Elsworth B (2018). The MR-Base platform supports systematic causal inference across the human phenome. Elife.

[25] Weale ME, Barnes MR, Breen G (2010). Quality control for genome-wide association studies. Genetic variation: methods and protocols.

[26] Fraser A, Macdonald-Wallis C, Tilling K (2013). Cohort profile: the Avon Longitudinal Study of Parents and Children: ALSPAC mothers cohort. Int J Epidemiol.

[27] Boyd A, Thomas R, Hansell AL (2019). Data resource profile: the ALSPAC birth cohort as a platform to study the relationship of environment and health and social factors. Int J Epidemiol.

[28] Relton CL, Gaunt T, McArdle W (2015). Data resource profile: Accessible Resource for Integrated Epigenomic Studies (ARIES). Int J Epidemiol.

[29] Min JL, Hemani G, Hannon E (2021). Genomic and phenotypic insights from an atlas of genetic effects on DNA methylation. Nat Genet.

[30] Timpson NJ, Nordestgaard BG, Harbord RM (2011). C-reactive protein levels and body mass index: elucidating direction of causation through reciprocal Mendelian randomization. Int J Obes (Lond).

[31] Machiela MJ, Chanock SJ (2015). LDlink: a web-based application for exploring population-specific haplotype structure and linking correlated alleles of possible functional variants. Bioinformatics.

[32] Wood AR, Tyrrell J, Beaumont R (2016). Variants in the FTO and CDKAL1 loci have recessive effects on risk of obesity and type 2 diabetes, respectively. Diabetologia.

[33] Shabalin AA (2012). Matrix eQTL: ultra fast eQTL analysis via large matrix operations. Bioinformatics.

[34] Sanderson E, Glymour MM, Holmes MV (2022). Mendelian randomization. Nat Rev Methods Primers.

[35] Hemani G, Tilling K, Davey Smith G (2017). Orienting the causal relationship between imprecisely measured traits using GWAS summary data. PLoS Genet.

[36] Bonder MJ, Luijk R, Zhernakova DV (2017). Disease variants alter transcription factor levels and methylation of their binding sites. Nat Genet.

[37] Battram T, Yousefi P, Crawford G (2022). The EWAS Catalog: a database of epigenome-wide association studies. Wellcome Open Res.

[38] Elliott HR (2021) Collapse EWAS catalog categories. Available from https://github.com/hannah-e/collapse_EWAS_catalog_phenotypes/blob/9b65be66399d0c1d2fd71c2003dbf58e4e5b62ff/functional_analysis_regroup_EWAS_catalogue_phenotypes.R. Accessed 25 July 2022

[39] Ochoa-Rosales C, Portilla-Fernandez E, Nano J (2020). Epigenetic link between statin therapy and type 2 diabetes. Diabetes Care.

[40] Dekkers KF, van Iterson M, Slieker RC (2016). Blood lipids influence DNA methylation in circulating cells. Genome Biol.

[41] Braun KVE, Dhana K, de Vries PS (2017). Epigenome-wide association study (EWAS) on lipids: the Rotterdam Study. Clin Epigenetics.

[42] Bohlin J, Håberg SE, Magnus P (2016). Prediction of gestational age based on genome-wide differentially methylated regions. Genome Biol.

[43] Küpers LK, Monnereau C, Sharp GC (2019). Meta-analysis of epigenome-wide association studies in neonates reveals widespread differential DNA methylation associated with birthweight. Nat Commun.

[44] Hedman ÅK, Mendelson MM, Marioni RE (2017). Epigenetic patterns in blood associated with lipid traits predict incident coronary heart disease events and are enriched for results from genome-wide association studies. Circ Cardiovasc Genet.

[45] Liu J, Carnero-Montoro E, van Dongen J (2019). An integrative cross-omics analysis of DNA methylation sites of glucose and insulin homeostasis. Nat Commun.

[46] Wahl S, Drong A, Lehne B (2017). Epigenome-wide association study of body mass index, and the adverse outcomes of adiposity. Nature.

[47] Demerath EW, Guan W, Grove ML (2015). Epigenome-wide association study (EWAS) of BMI, BMI change and waist circumference in African American adults identifies multiple replicated loci. Hum Mol Genet.

[48] Klimentidis YC, Arora A, Newell M (2020). Phenotypic and genetic characterization of lower LDL cholesterol and increased type 2 diabetes risk in the UK Biobank. Diabetes.

[49] Fall T, Xie W, Poon W (2015). Using genetic variants to assess the relationship between circulating lipids and type 2 diabetes. Diabetes.

[50] Elliott HR, Burrows K, Min JL (2022). Characterisation of ethnic differences in DNA methylation between UK-resident South Asians and Europeans. Clin Epigenetics.

