# Supplementary material for: Investigating causality in the association between DNA methylation and type 2 diabetes using bidirectional two-sample Mendelian randomisation
Source: Diabetologia. 2023 May 19;66(7):1247–59. doi: 10.1007/s00125-023-05914-7 (PMC10244277; doi:10.1007/s00125-023-05914-7)
Supplement: Supplementary file 1 — Supplementary file1 (PDF 1577 KB) [file 125_2023_5914_MOESM1_ESM.pdf]

Electronic Supplementary material (ESM)

ESM Methods S1. ALSPAC ARIES

Pregnant women resident in Avon, UK with expected dates of delivery 1st April 1991 to 31st December 1992 were invited to take part in the study. The initial number of pregnancies enrolled is 14,541 (for these at least one questionnaire has been returned or a "Children in Focus" clinic had been attended by 19/07/99). Of these initial pregnancies, there was a total of 14,676 foetuses, resulting in 14,062 live births and 13,988 children who were alive at 1 year of age (1, 2). When the oldest children were approximately 7 years of age, an attempt was made to bolster the initial sample with eligible cases who had failed to join the study originally. As a result, when considering variables collected from the age of seven onwards (and potentially abstracted from obstetric notes) there are data available for more than the 14,541 pregnancies mentioned above. The number of new pregnancies not in the initial sample (known as Phase I enrolment) that are currently represented on the built files and reflecting enrolment status at the age of 24 is 913 (456, 262 and 195 recruited during Phases II, III and IV respectively), resulting in an additional 913 children being enrolled. The phases of enrolment are described in more detail in the cohort profile paper and its update (1, 2). The total sample size for analyses using any data collected after the age of seven is therefore 15,454 pregnancies, resulting in 15,589 foetuses. Of these 14,901 were alive at 1 year of age. A 10% sample of the ALSPAC cohort, known as the Children in Focus (CiF) group, attended clinics at the University of Bristol at various time intervals between 4 to 61 months of age. The CiF group were chosen at random from the last 6 months of ALSPAC births (1432 families attended at least one clinic) (1, 2). Excluded were those mothers who had moved out of the area or were lost to follow-up, and those partaking in another study of infant development in Avon. In our MR study of T2D, we considered a subsample of 867 mothers and 385 fathers from the main ALSPAC cohort with genetic and epigenetic (DNA methylation) data to conduct analyses.

Please note that the study website contains details of all the data that is available through a fully searchable data dictionary and variable search tool (http://www.bristol.ac.uk/alspac/researchers/our-data/)

Ethical approval for the study was obtained from the ALSPAC Ethics and Law Committee and the Local Research Ethics Committees. Consent for biological samples has been collected in accordance with the Human Tissue Act (2004).

ESM Methods S2. Selecting genetic proxies for meta-EWAS CpGs in GoDMC

To assess if increased DNAm at selected meta-EWAS CpGs was causal of T2D risk, we searched for genetic proxies for 58 meta-EWAS of T2D CpGs in the GoDMC consortium. GoDMC is currently the biggest consortium for the study of the genetics of DNAm variation, comprising 32,851 samples across 36 contributing cohorts (3). Samples included in GoDMC were mostly of European ancestry, with mean age of 55.6 years. In the discovery stage, around 248,607 independent *cis*-mQTL (SNP < 1 Mb from CpG site) and 23,117 *trans*-mQTL (SNP > 1 Mb from CpG site) were reported using a p-threshold $< 10^{-8}$ and $p < 10^{-14}$, respectively (3). Regression analyses between the genotype and DNAm were adjusted for age, sex, predicted cell counts, predicted smoking and genetic PCs among unrelated participants; for related participants in addition to the above, fitting was applied for the genetic kinship matrix using the method described in GRAMMAR (3). Additional information about GoDMC and their study design, can be found elsewhere (3).

Of the 58 T2D CpGs of interest, 31 had at least one mQTL reported in GoDMC. Of these 31 CpGs, 22 had one mQTL, 8 CpGs (in *HDAC4*, *ITIH1*, *PHGDH*, *ROBO1*, *SMYD5*, cg00896068, cg27037013 and cg27115863) had two mQTL, and the CpG in *DHCR24* had three mQTL reported in GoDMC. Association estimates between the mQTL SNPs and T2D were retrieved from two previously published GWAS in T2D (4, 5) included in the MR-Base database. From these two studies, we were able to successfully extract genetic association data with T2D for 39 mQTL SNPs typing 30 meta-EWAS CpGs after data harmonization in MR-Base. We excluded the mQTL SNP rs74623153 (typing cg11983038) without GWAS information, and rs9976794 (typing cg27037013) that was a palindromic SNP with intermediate allele frequencies.

ESM Methods S3. Mendelian Randomisation and MR-Base analysis

Mendelian randomisation (MR) is an analytical method used to investigate the plausibility of causality from observational associations. To assess causality, MR relies on genetic variants as natural experiments to predict the unbiased association between a modifiable exposure and an outcome. As any other method, MR relies on three core assumptions: i) the genetic instruments are strongly associated with the exposure, ii) the association of the genetic instruments with the outcome is solely via the exposure, iii) the genetic instruments are not related to the confounders (6).

To conduct the two-sample MR analysis, we implemented tools available in the TwoSampleMR (version 0.5.6), MRInstruments (version 0.3.2) and MendelianRandomization (version 0.5.1) R packages to conduct causal analyses (7, 8). We applied *clumping* to select only independent instruments (LD $r^2 < 0.01$) for T2D and DNAm. For the causal analysis, we used default parameters in MR-Base and selected as our main findings results of the inverse-variance weighted (IVW) regression using a random-effect model, or those from a Wald ratio when the number of instruments available was < 2 SNPs. When there were > 2 instruments, we included three sensitivity analyses for comparison with results of the IVW: the MR-Egger regression to account for horizontal pleiotropy in the effect of proxy SNPs on the outcome (8), and the weighted median and weighted mode regressions to account for potential invalid instruments (7, 8). Evidence of directional pleiotropy was considered if the intercept of the MR-Egger regression had a $p < 0.001$ ($\alpha = 0.05/62$ T2D SNPs). We applied correction for multiple testing in results of the MR analysis using an $\alpha = 0.05/58$ CpGs or $p < 1.0\text{x}10^{-3}$ for the forward MR (T2D → 58 meta-EWAS CpGs), or an $\alpha = 0.05/30$ CpGs or $p < 2.0\text{x}10^{-3}$ for the reverse MR (30 successfully proxied meta-EWAS CpGs → T2D). We deemed as suggestive of causality associations with an uncorrected $p < 0.05$.

We measured SNP heterogeneity using the Cochran's Q estimate derived from the IVW, and the Rucker's Q estimate obtained from the MR-Egger regression. Heterogeneity was significant if $Q > 70$ and $p < 0.001$. We used the Steiger test to verify that true direction of association was the one specified in the analysis. This analysis may perform better when using continuous exposures (i.e., DNAm as opposed to T2D). Lastly, we implemented a leave-one-out analysis using the IVW method to identify potential outlier SNPs (7). Strength of the instruments was estimated in

each direction of the two-sample MR using the F-statistic reported by the IVW, or it was manually calculated as the beta-coefficient/standard error for the SNP-exposure association. We disregarded presence of weak instrument bias in MR findings if the F-statistic > 10. When possible, different graphical methods, like scatter plots, funnel plots, forest plots and volcano plots, were used to represent results of the MR analysis and validate MR assumptions.

ESM Methods S4. Extraction of genotype data from ALSPAC-ARIES samples

Genotyping was completed prior to this project. Thus, for middle-aged adults in ALSPAC, genetic data for 148 T2D SNPs was extracted from the ALSPAC GWAS database (1) using the latest genetic imputation datasets available (mothers: release 2015-10-30, fathers: release 2016-11-22). Because imputation was done independently for mothers and fathers in ALSPAC, both datasets were merged retaining the first ten genetic principal components (PCs) to adjust for genetic structure in downstream analyses. Detail of the method applied for genotyping and imputation of genetic data in mothers in ALSPAC has been described elsewhere (1, 9). Briefly, genetic data was generated using the Illumina Infinium Human660W-Quad BeadChip array v1.0, and the Illumina GenomeStudio software for genotyping calling (genome build 37). Quality control (QC) measures applied included removal of SNPs with minor allele frequency (MAF) < 0.01, Hardy-Weinberg equilibrium p-value < 10^{-6}, and missing genotyping rate > 0.05. In addition, samples with indeterminate X chromosome heterozygosity, genotyping missingness higher than 5%, and evidence of population stratification, were excluded. Data was imputed to the 1,000 Genomes (phase 1, version 3, http://www.internationalgenome.org/about) using Impute2 version 2.2.2, retaining samples with MAF > 0.01 and calling rate > 80%.

Genetic data for the fathers was genotyped using the Illumina HumanCoreExome BeadChip array, and the Illumina GenomeStudio software for variant calling. QC measures included removing SNPs with Hardy-Weinberg equilibrium p-value < 10^{-7}, SNPs failing GenomeStudio QC, and SNPs that were duplicated. Samples with gender mismatch, high or low heterozygosity, genotyping missingness > 5%, contamination, and of non-European origin, were excluded. Before imputation, genotype data for 3,074 fathers (some of them related) was furtherly controlled for variants not included in the 1,000 Genomes, monomorphic SNPs and duplicated

sites. Imputation was performed as in the mother's dataset. Data from the imputation was retained if MAF > 0.01 and calling rate > 80%. For consistency, SNP annotations use genome build 37 throughout.

ESM Methods S5. Quality control of genetic data in ALSPAC-ARIES

Imputed genetic data extracted for middle-aged adults in ALSPAC-ARIES was QC before the analysis. Imputed genotype data was available for 6,102,837 SNPs after merging the genetic datasets of mothers and fathers in ALSPAC using a consensus method in Plink. Genotyping rate for these variants was 0.98, and further inspection of the data included plotting imputation quality scores against the MAF to verify that variants remaining in the dataset had MAF > 0.01, and high imputation rate (info > 0.86). Additional QC for the SNPs included removing those with MAF < 0.01, calling rate < 0.8, missing genotyping rate > 0.05, and Hardy-Weinberg equilibrium p-value < 10^{-6}. Samples were excluded if cryptic relatedness between pairwise comparisons was higher than 0.125, and if missing genotyping rate was > 0.05. Twenty PCs were generated for the genotype data of unrelated samples using a list of 26,873 independent SNPs identified in the HapMap3 project with Linkage Disequilibrium or LD < 0.1 and MAF > 0.2. None of the samples were identified as an outlier based on genetic variation measured by the PCs. Twenty genetic PCs were used as covariates in processing of the DNA methylation data. In total, 1,243 middle-aged adults and approximately 5.3 million SNPs remained in the dataset after QC. Total genotyping rate was 0.99, average missing genotyping rate was 0.01, and correlation between observed and expected allele frequencies in the 1,000 Genomes was 0.99.

ESM Methods S6. Quality control of DNA methylation data in ALSPAC-ARIES

Genome-wide DNA methylation was available for 482,015 probes, including probes in sex chromosomes and probes reported in the Naeem list (10). For consistency, CpG annotations use genome build 37 throughout. Genomic features and proximity to RefSeq genes were annotated using *Meffil*. Methylation data was initially QC for outliers, which were identified as samples with methylation levels ten standard deviations higher than the mean of the probe. Outliers were detected after three iterations and replaced by the mean of the probe.

Normalization of the methylation data was applied to avoid false positives in the SNP-CpG analysis, and to minimize the amount of non-genetic residual variation remaining in the data. To achieve this, methylation was first inverse-normal transformed, and then regressed against covariates and twenty genetic PCs previously generated (see section S3). We obtained residuals of DNAm using a regression model adjusted for age, sex, batch effects (i.e., bisulphite conversion plate), predicted counts for seven white-blood cells (Houseman method), and predicted levels of smoking. Predicted levels of smoking were estimated using data from two DNAm scores developed by Zeilinger *et al.* (11) and by Elliott *et al.* (12). Because only complete data was required in the dataset of covariates, missing data for age in a subset of males was replaced using predictive mean matching imputation method in the R package MICE (13). Covariates and genetic PCs were regarded as fixed effects in the regression model. Residuals of this analysis were then regressed against methylation PCs estimated to account for residual confounding. Methylation PCs were generated using the 20,000 most variable autosomal probes, and they were tested for association with the genotype (i.e., 62 T2D SNPs) using a linear regression model in the MatrixeQTL R package. Methylation PCs associated with the genotype at *p*-value < 10^{-7} were excluded. In total, the first thirteen non-genetic methylation PCs were retained for further analyses. Residuals of the regression between adjusted methylation values at 482,015 probes and methylation PCs, were used in the SNP-CpG analysis. The linear regression model used for analysis in MatrixeQTL was the following:

$$\text{CpGn} = \alpha + \Sigma_k\, \beta_k \cdot \text{covariate}_k + \gamma \cdot \text{genotype\_additive}$$

Where β_k and γ are the effect of covariates and the genotype (i.e., per effect allele) on variation in DNA methylation, respectively.

ESM Methods S7. Results of sensitivity analyses implemented in the forward 2SMR (T2D → DNAm)

When we applied additional MR methods with less stringent assumptions about the validity of the instruments like the weighted median and weighted mode analyses, they showed same direction of association and similar *p*-value as results of the IVW at top two CpGs identified in the forward 2SMR, cg20812370 (*PBX1*) and cg01577083 (*RBFOX1*). However, absolute effect

estimates were slightly larger when using these other methods. Even though we found no evidence of pleiotropy in the association at cg20812370 (*PBX1*) [Cochran's Q estimate 52.9, *P*= 0.76], we observed some asymmetry in the funnel plot for this site attributed to two outlier SNPs, rs319598 and rs1359790, which in the forest plot were also seen with a more negative effect in cg20812370 (*PBX1*) compared to the rest of the SNPs (ESM Figure 1). We re-rn the analysis for this CpG excluding the two outlier SNPs, and we observed a less negative effect estimate but a larger *p*-value this time around (p = 0.002 with and p = 0.01 without outlier SNPs). Applying a leave-one-out analysis to investigate the effect of influential SNPs on the outcome (DNAm), we did not find evidence that any single T2D SNP was independently driving the total causal estimate at cg20812370 (*PBX1*) or at cg01577083 (*RBFOX1*). The Steiger test suggested that true direction of association at cg01577083 was from DNAm to T2D, and not the direction here analyzed (Steiger p=5.5x10^{-5}, R^2 for T2D = 0.03 < R^2 for cg01577083 = 0.07). Summary plots of the forward 2SMR for cg20812370 (*PBX1*) and cg01577083 (*RBFOX1*) can be found in ESM Figures 1-2.

ESM Table 1. Four GWAS of T2D reported in the DIAGRAM consortium and used to extract genetic proxies for T2D in the forward two-sample MR analysis (T2D → meta-EWAS CpGs).

SNPs	Data source	Population	Discovery sample	Replication sample	Notes
60	Morris *et al.* 2012<br>PMID: 22885922	Europeans	121,171 cases and 56,862 controls	22,669 cases and 58,119 controls	Genotyping method was the Metabochip array. Meta-analysis of GWAS adjusted for genomic inflation
		Pakistani (PROMIS)		1,178 cases and 2,472 controls	
34	Mahajan *et al.* 2014<br>PMID: 24509480	Europeans	12,171 cases and 56,862 controls	21,491 cases and 55,647 controls	Ancestry-specific GWAS corrected for study-specific covariates and genomic inflation. Trans-ethnic meta-analysis corrected for genomic inflation.
		South Asians	6,952 cases and 11,865 controls		
		East Asians	5,561 cases and 14,458 controls		
		Mexicans and Mexican Americans	1,804 cases and 779 controls		
40	Gaulton *et al.* 2015<br>PMID: 26551672	Europeans	27,206 cases and 57,574 controls	Not reported	Fine mapping of 39 established genetic loci in T2D
14	Fuchsberger *et al.* 2016<br>PMID: 27398621	Europeans<br>East Asians<br>South Asians<br>African<br>American Hispanics	11,645 cases and 32,769 controls	Not reported	

ESM Table 2. Association estimates of 62 SNPs extracted from the DIAGRAM consortium and used as genetic proxies for T2D in the forward two-sample MR analysis (T2D → meta-EWAS CpGs). Associations are ordered by *p*-value (from smallest to largest).

SNP	Chr	Mapped gene	Estimate	SE	EA	OA	EAF	*p*	N
rs7903146	10	*TCF7L2*	0.33	0.02	T	C	0.26	1.20E-139	144,178
rs10811660	9	*Unannotated*	0.24	0.02	G	A	0.83	1.10E-61	219,582
rs35261542	6	*CDKAL1*	0.16	0.01	A	C	0.28	1.50E-50	219,582
rs35510946	3	*IGF2BP2*	0.13	0.01	A	G	0.3	1.10E-39	219,582
rs11187140	10	*Unannotated*	0.11	0.01	G	A	0.63	1.50E-31	219,582
rs13266634	8	*SLC30A8*	0.11	0.01	C	T	0.68	5.00E-28	219,582
rs1513272	7	*JAZF1*	0.1	0.01	C	T	0.52	7.80E-25	219,582
rs9936385	16	*FTO*	0.12	0.02	C	T	0.40	2.60E-23	144,178
rs11712037	3	*PPARG*	0.13	0.02	C	G	0.86	1.70E-20	219,582
rs2972156	2	*Unannotated*	0.09	0.01	G	C	0.62	4.20E-20	219,582
rs11257658	10	*Unannotated*	0.09	0.01	A	G	0.22	1.20E-15	219,582
rs72999033	19	*HAPLN4*	0.15	0.02	T	C	0.07	1.80E-15	219,582
rs7607980	2	*COBLL1*	0.14	0.02	T	C	0.88	8.30E-15	92,794
rs6813195	4	*RPS3AP18; RPS14P6*	0.08	0.01	C	T	0.73	4.10E-14	161,639
rs77981966	2	*THADA*	0.15	0.02	C	T	0.93	4.10E-14	219,582
rs11717195	3	*ADCY5*	0.1	0.02	T	C	0.78	6.50E-14	149,821
rs340874	1	*PROX1*	0.07	0.01	C	T	0.52	5.10E-13	219,582
rs7732130	5	*ZBED3-AS1*	0.08	0.01	G	A	0.28	2.40E-12	219,582
rs17676309	3	*ADAMTS9-AS2; MIR548A2*	0.07	0.01	C	T	0.59	2.80E-12	219,582
rs1387153	11	*MTNR1B*	0.09	0.02	T	C	0.29	1.60E-11	144,178
rs2583941	12	*RPSAP52*	0.1	0.02	A	G	0.09	1.60E-11	219,582
rs10276674	7	*DGKB*	0.08	0.01	C	T	0.18	2.80E-11	219,582
rs3803563	15	*PRC1*	0.08	0.01	A	C	0.18	5.60E-11	219,582
rs12571751	10	*ZMIZ1*	0.08	0.01	A	G	0.51	1.00E-10	149,821
rs878521	7	*YKT6; CAMK2B*	0.07	0.01	A	G	0.24	1.30E-10	219,582
rs1552224	11	*ARAP1*	0.1	0.02	A	C	0.83	1.80E-10	144,178
rs516946	8	*ANK1; MIR486*	0.09	0.02	C	T	0.77	2.50E-10	149,821
rs35720761	2	*THADA*	0.11	0.02	T	C	0.89	3.30E-10	92,794
rs780094	2	*GCKR*	0.06	0.01	C	T	0.61	3.40E-10	219,582
rs243020	2	*Unannotated*	0.06	0.01	G	A	0.46	5.50E-10	219,582
rs35658696	5	*PAM*	0.16	0.03	A	G	0.96	5.70E-10	92,794
rs10842994	12	*KLHL42; PTHLH*	0.1	0.02	C	T	0.8	6.10E-10	149,821
rs5215	11	*KCNJ11*	0.07	0.01	C	T	0.39	8.50E-10	149,821
rs1974620	7	*Unannotated*	0.06	0.01	T	C	0.52	1.00E-09	219,582
rs1496653	3	*UBE2E2; MIR548AC*	0.09	0.02	A	G	0.79	3.60E-09	149,821

ESM Table 2. (*Continued*)

SNP	Chr	Mapped gene	Estimate	SE	EA	OA	EAF	*p*	N
rs17106184	1	*FAF1*	0.1	0.02	G	A	0.91	4.10E-09	161,585
rs7177055	15	*Unannotated*	0.08	0.01	A	G	0.72	4.60E-09	149,821
rs2796441	9	*LOC101927502*	0.07	0.01	G	A	0.63	5.40E-09	147,724
rs41278853	22	*MTMR3*	0.13	0.03	A	G	0.89	5.60E-09	92,794
rs6808574	3	*BCL6; LPP-AS2*	0.07	0.01	C	T	0.6	5.80E-09	140,087
rs7955901	12	*Unannotated*	0.07	0.01	C	T	0.42	6.50E-09	144,178
rs702634	5	*ARL15*	0.06	0.01	A	G	0.71	6.90E-09	154,797
rs4275659	12	*ABCB9*	0.06	0.01	C	T	0.67	9.50E-09	161,459
rs12970134	18	*RPS3AP49; MC4R*	0.08	0.02	A	G	0.26	1.20E-08	138,946
rs1359790	13	*LINC01080; SPRY2*	0.08	0.01	G	A	0.73	1.40E-08	149,821
rs7202877	16	*CTRB1-CTRB2*	0.11	0.02	T	G	0.9	3.50E-08	144,178
rs4812829	20	*HNF4A*	0.07	0.03	A	G	0.16	5.00E-08	77,138
rs7845219	8	*CCNE2; TP53INP1*	0.08	0.02	T	C	0.53	6.00E-08	77,138
rs7961581	12	*TSPAN8*	0.06	0.01	C	T	0.27	1.80E-07	219,582
rs10190052	2	*FAM150B; TMEM18*	0.07	0.02	C	T	0.87	2.00E-07	77,138
rs9472138	6	*TRNAI25*	0.06	0.01	T	C	0.25	2.00E-07	77,138
rs2028299	15	*AP3S2; C15orf38-AP3S2*	0.04	0.02	C	A	0.29	5.00E-07	77,138
rs2820446	1	*RIMKLBP2; ZC3H11B*	0.05	0.01	C	G	0.72	2.00E-06	77,138
rs319598	5	*PCBD2*	0.05	0.01	C	T	0.53	2.00E-06	77,138
rs4273712	6	*YAP1P3; PRELID1P1*	0.05	0.01	G	A	0.25	3.00E-06	77,138
rs12427353	12	*HNF1A*	0.11	0.03	G	C	0.79	4.00E-06	77,138
rs7041847	9	*GLIS3*	0.05	0.02	A	G	0.51	5.00E-06	77,138
rs6937795	6	*SLC35D3; RPL35AP3*	0.04	0.01	A	C	0.42	7.00E-06	77,138
rs1535500	6	*KCNK16; KCNK17*	0.12	0.03	T	G	0.59	8.00E-06	77,138
rs2284219	7	*CRHR2*	0.05	0.01	G	A	0.66	8.00E-06	77,138
rs10788575	10	*RPL11P3; MED6P1*	0.06	0.01	A	G	0.17	9.00E-06	77,138
rs16861329	3	*ST6GAL1*	0.03	0.04	C	T	0.85	9.00E-06	77,138

Estimate: log(odds) of T2D per increase in the effect allele, EA: effect allele, OA: other allele, EAF: effect allele frequency, N: sample-size.

ESM Table 3. Association between T2D SNPs and CpGs identified in a meta-EWAS of prevalent T2D, using ALSPAC-ARIES samples (N=1,243). SNP-CpG pairs shown were identified with uncorrected *p* < 0.05 (Bonferroni p < 1.4x10^{-5} or α= 0.05/62 SNPs * 58 CpGs). Associations are ordered by *p*-value (from smallest to largest).

SNP	CpG	SNP gene	Estimate	SE	EA	OA	EAF	*p*	N
rs4275659	cg10584271	ABCB9	0.225	0.045	T	C	0.702	5.3E-07	1216
rs9936385	cg14284506	FTO	0.158	0.041	C	T	0.623	1.5E-04	1242
rs12427353	cg08945443	HNF1A	-0.178	0.051	C	G	0.804	5.5E-04	1201
rs1359790	cg20812370	SPRY2	0.161	0.047	A	G	0.725	5.8E-04	1216
rs780094	cg15832662	GCKR	-0.136	0.040	T	C	0.586	7.5E-04	1243
rs2284219	cg00574958	CRHR2	0.139	0.042	A	G	0.650	8.6E-04	1233
rs944801	cg16192197	NA	0.135	0.040	G	C	0.574	8.8E-04	1219
rs13266634	cg04567334	SLC30A8	-0.138	0.043	T	C	0.685	1.3E-03	1243
rs2284219	cg00989505	CRHR2	0.130	0.042	A	G	0.650	1.8E-03	1233
rs5215	cg15560632	KCNJ11	0.128	0.041	C	T	0.630	2.1E-03	1243
rs3803563	cg15832662	PRC1	-0.164	0.053	A	C	0.834	2.1E-03	1236
rs11712037	cg01577083	PPARG	-0.189	0.062	G	C	0.886	2.3E-03	1233
rs4275659	cg14003143	NA	0.131	0.045	T	C	0.702	3.6E-03	1216
rs72999033	cg14476101	HAPLN4	0.233	0.080	T	C	0.935	3.8E-03	1210
rs1496653	cg11851382	NA	-0.139	0.048	G	A	0.782	4.0E-03	1243
rs17106184	cg01963618	FAF1	-0.192	0.067	A	G	0.907	4.2E-03	1236
rs6808574	cg20116935	LPP	0.116	0.041	T	C	0.623	4.6E-03	1198
rs35510946	cg20316538	IGF2BP2	0.122	0.043	A	G	0.692	4.8E-03	1229
rs7903146	cg20456243	TCF7L2	-0.122	0.044	T	C	0.703	5.3E-03	1243
rs7177055	cg00162348	NA	0.125	0.045	G	A	0.720	5.4E-03	1242
rs516946	cg11376147	ANK1	0.129	0.046	T	C	0.753	5.5E-03	1232
rs7961581	cg00574958	TSPAN8;LGR5	0.125	0.045	C	T	0.735	5.5E-03	1235
rs10811660	cg11851382	NA	0.141	0.051	A	G	0.818	5.6E-03	1222
rs702634	cg24704287	ARL15	0.124	0.045	G	A	0.709	5.7E-03	1213
rs6937795	cg15832662	IL20RA	-0.110	0.040	C	A	0.521	6.5E-03	1223
rs516946	cg16765088	ANK1	0.126	0.046	T	C	0.753	6.6E-03	1232
rs72999033	cg27374726	HAPLN4	-0.218	0.080	T	C	0.935	6.7E-03	1210
rs4275659	cg07184465	NA	0.122	0.045	T	C	0.702	6.7E-03	1216
rs340874	cg07068382	PROX1	0.110	0.041	T	C	0.577	7.1E-03	1242
rs7041847	cg20154947	GLIS3	-0.107	0.040	T	C	0.522	8.1E-03	1236
rs10811660	cg25536676	NA	-0.134	0.051	A	G	0.818	8.6E-03	1222
rs340874	cg07184465	PROX1	0.107	0.041	T	C	0.577	8.8E-03	1242
rs2820446	cg15832662	LYPLAL1	-0.116	0.044	G	C	0.704	8.9E-03	1238
rs1974620	cg19876302	NA	-0.103	0.040	C	T	0.528	9.3E-03	1243

ESM Table 3. (*Continued*)

SNP	CpG	SNP gene	Estimate	SE	EA	OA	EAF	*p*	N
rs6937795	cg18181703	IL20RA	0.105	0.040	C	A	0.521	9.5E-03	1223
rs11712037	cg11851382	PPARG	0.160	0.062	G	C	0.886	9.5E-03	1233
rs77981966	cg18181703	THADA	0.203	0.078	T	C	0.928	9.6E-03	1206
rs72999033	cg08857797	HAPLN4	0.208	0.080	T	C	0.935	9.7E-03	1210
rs7903146	cg01577083	TCF7L2	-0.113	0.044	T	C	0.703	9.8E-03	1243
rs4275659	cg24512093	NA	0.116	0.045	T	C	0.702	1.0E-02	1216
rs7607980	cg00989505	COBLL1	-0.148	0.058	C	T	0.863	1.0E-02	1242
rs1535500	cg12593793	KCNK16	0.100	0.039	T	G	0.522	1.1E-02	1229
rs7961581	cg14476101	TSPAN8;LGR5	-0.114	0.045	C	T	0.735	1.1E-02	1235
rs10842994	cg24704287	KLHDC5	0.128	0.051	T	C	0.804	1.2E-02	1234
rs7041847	cg11024682	GLIS3	-0.102	0.040	T	C	0.522	1.2E-02	1236
rs340874	cg00144180	PROX1	0.103	0.041	T	C	0.577	1.2E-02	1242
rs319598	cg20116935	PCBD2	0.104	0.042	T	C	0.582	1.2E-02	1237
rs5215	cg14476101	KCNJ11	-0.104	0.042	C	T	0.630	1.2E-02	1243
rs319598	cg20316538	PCBD2	0.103	0.042	T	C	0.582	1.4E-02	1237
rs5215	cg08857797	KCNJ11	-0.102	0.042	C	T	0.630	1.4E-02	1243
rs11257658	cg06039489	NA	-0.121	0.049	A	G	0.776	1.4E-02	1219
rs2972156	cg08857797	NA	-0.102	0.042	C	G	0.629	1.4E-02	1221
rs35720761	cg11252555	THADA	-0.146	0.060	T	C	0.871	1.5E-02	1236
rs7732130	cg00144180	ZBED3-AS1	0.108	0.044	G	A	0.697	1.5E-02	1207
rs5215	cg00896068	KCNJ11	-0.101	0.042	C	T	0.630	1.5E-02	1243
rs10510110	cg01577083	PLEKHA1	-0.098	0.040	C	T	0.526	1.5E-02	1236
rs702634	cg00574958	ARL15	-0.109	0.045	G	A	0.709	1.5E-02	1213
rs2796441	cg01577083	TLE1	-0.100	0.042	A	G	0.580	1.6E-02	1187
rs7955901	cg20316538	NA	0.098	0.041	C	T	0.569	1.6E-02	1240
rs4275659	cg27374726	NA	0.108	0.045	T	C	0.702	1.6E-02	1216
rs12571751	cg14476101	ZMIZ1	0.098	0.041	G	A	0.543	1.6E-02	1233
rs7161785	cg27374726	NA	-0.097	0.041	C	G	0.568	1.7E-02	1239
rs10811660	cg00896068	NA	-0.121	0.051	A	G	0.818	1.7E-02	1222
rs1513272	cg00896068	NA	-0.096	0.040	C	T	0.505	1.7E-02	1231
rs2796441	cg14284506	TLE1	-0.099	0.042	A	G	0.580	1.7E-02	1187
rs340874	cg27115863	PROX1	-0.097	0.041	T	C	0.577	1.7E-02	1242
rs35720761	cg08570691	THADA	-0.142	0.060	T	C	0.871	1.8E-02	1236
rs35658696	cg20154947	PAM	-0.225	0.095	G	A	0.952	1.8E-02	1194
rs1359790	cg16192197	SPRY2	-0.110	0.047	A	G	0.725	1.8E-02	1216
rs7955901	cg20231084	NA	0.096	0.041	C	T	0.569	1.8E-02	1240
rs340874	cg00162348	PROX1	-0.096	0.041	T	C	0.577	1.9E-02	1242
rs17106184	cg01577083	FAF1	0.158	0.067	A	G	0.907	1.9E-02	1236
rs7845219	cg01963618	TP53INP1	-0.093	0.039	T	C	0.511	1.9E-02	1229

ESM Table 3. (*Continued*)

SNP	CpG	SNP gene	Estimate	SE	EA	OA	EAF	*p*	N
rs17106184	cg07212837	FAF1	0.157	0.067	A	G	0.907	1.9E-02	1236
rs7607980	cg24795867	COBLL1	-0.135	0.058	C	T	0.863	2.0E-02	1242
rs9936385	cg08570691	FTO	-0.097	0.042	C	T	0.623	2.0E-02	1242
rs2972156	cg06039489	NA	0.097	0.042	C	G	0.629	2.0E-02	1221
rs516946	cg25741837	ANK1	-0.108	0.046	T	C	0.753	2.0E-02	1232
rs1974620	cg24704287	NA	-0.092	0.040	C	T	0.528	2.0E-02	1243
rs7845219	cg11024682	TP53INP1	0.092	0.039	T	C	0.511	2.0E-02	1229
rs7955901	cg20154947	NA	-0.094	0.041	C	T	0.569	2.1E-02	1240
rs77981966	cg16765088	THADA	0.182	0.079	T	C	0.928	2.1E-02	1206
rs10190052	cg01963618	TMEM18	-0.118	0.051	T	C	0.813	2.1E-02	1243
rs35261542	cg14284506	CDKAL1	-0.106	0.046	A	C	0.740	2.1E-02	1229
rs340874	cg07212837	PROX1	0.094	0.041	T	C	0.577	2.1E-02	1242
rs1496653	cg20812370	NA	0.110	0.048	G	A	0.782	2.2E-02	1243
rs12571751	cg08570691	ZMIZ1	0.093	0.041	G	A	0.543	2.2E-02	1233
rs243020	cg07212837	NA	-0.090	0.040	G	A	0.540	2.3E-02	1238
rs1974620	cg14284506	NA	-0.090	0.040	C	T	0.528	2.3E-02	1243
rs9472138	cg11983038	VEGFA	-0.101	0.045	T	C	0.712	2.3E-02	1243
rs4275659	cg20231084	NA	0.102	0.045	T	C	0.702	2.3E-02	1216
rs5215	cg15832662	KCNJ11	-0.093	0.042	C	T	0.630	2.5E-02	1243
rs1513272	cg01577083	NA	-0.090	0.040	C	T	0.505	2.6E-02	1231
rs11712037	cg14476101	PPARG	0.138	0.062	G	C	0.886	2.6E-02	1233
rs2028299	cg06500161	AP3S2	0.098	0.044	C	A	0.704	2.6E-02	1235
rs878521	cg27374726	YKT6	-0.106	0.048	A	G	0.751	2.6E-02	1202
rs35261542	cg16575444	CDKAL1	0.102	0.046	A	C	0.740	2.6E-02	1229
rs2972156	cg11851382	NA	0.092	0.042	C	G	0.629	2.7E-02	1221
rs10276674	cg20456243	NA	-0.118	0.053	C	T	0.816	2.7E-02	1203
rs7041847	cg10082515	GLIS3	-0.089	0.040	T	C	0.522	2.8E-02	1236
rs35720761	cg04567334	THADA	-0.131	0.060	T	C	0.871	2.9E-02	1236
rs10788575	cg19693031	PTEN	-0.117	0.054	A	G	0.835	2.9E-02	1236
rs7955901	cg00574958	NA	0.089	0.041	C	T	0.569	2.9E-02	1240
rs1552224	cg00896068	ARAP1	0.118	0.054	C	A	0.836	2.9E-02	1243
rs1387153	cg00896068	MTNR1B	0.096	0.044	T	C	0.707	2.9E-02	1243
rs7041847	cg14003143	GLIS3	0.088	0.040	T	C	0.522	2.9E-02	1236
rs35510946	cg11024682	IGF2BP2	0.094	0.043	A	G	0.692	3.0E-02	1229
rs12970134	cg07184465	MC4R	0.099	0.046	A	G	0.739	3.0E-02	1243
rs944801	cg20231084	NA	-0.088	0.041	G	C	0.574	3.0E-02	1219
rs13266634	cg13178597	SLC30A8	0.093	0.043	T	C	0.685	3.1E-02	1243
rs6808574	cg06500161	LPP	0.089	0.041	T	C	0.623	3.1E-02	1198
rs9472138	cg06114363	VEGFA	0.096	0.045	T	C	0.712	3.1E-02	1243

ESM Table 3. (*Continued*)

SNP	CpG	SNP gene	Estimate	SE	EA	OA	EAF	*p*	N
rs9936385	cg11024682	FTO	0.090	0.042	C	T	0.623	3.1E-02	1242
rs944801	cg00896068	NA	-0.087	0.041	G	C	0.574	3.1E-02	1219
rs35658696	cg27374726	PAM	0.205	0.095	G	A	0.952	3.2E-02	1194
rs17106184	cg11376147	FAF1	0.144	0.067	A	G	0.907	3.2E-02	1236
rs10842994	cg27037013	KLHDC5	0.109	0.051	T	C	0.804	3.2E-02	1234
rs12427353	cg12593793	HNF1A	0.110	0.052	C	G	0.804	3.2E-02	1201
rs878521	cg14284506	YKT6;CAMK2B	0.102	0.048	A	G	0.751	3.2E-02	1202
rs2028299	cg26270261	AP3S2	0.094	0.044	C	A	0.704	3.2E-02	1235
rs4273712	cg15832662	C6orf173	0.095	0.044	G	A	0.719	3.3E-02	1243
rs243020	cg22680424	NA	-0.085	0.040	G	A	0.540	3.3E-02	1238
rs77981966	cg11252555	THADA	-0.168	0.079	T	C	0.928	3.3E-02	1206
rs17106184	cg00989505	FAF1	-0.143	0.067	A	G	0.907	3.3E-02	1236
rs7961581	cg18181703	TSPAN8;LGR5	0.096	0.045	C	T	0.735	3.3E-02	1235
rs35720761	cg18181703	THADA	0.127	0.060	T	C	0.871	3.3E-02	1236
rs4275659	cg20316538	NA	0.096	0.045	T	C	0.702	3.3E-02	1216
rs319598	cg20812370	PCBD2	0.089	0.042	T	C	0.582	3.3E-02	1237
rs1387153	cg25741837	MTNR1B	0.093	0.044	T	C	0.707	3.4E-02	1243
rs12571751	cg20316538	ZMIZ1	-0.087	0.041	G	A	0.543	3.4E-02	1233
rs10190052	cg25136644	TMEM18	0.108	0.051	T	C	0.813	3.4E-02	1243
rs1552224	cg06114363	ARAP1,CENTD2	0.115	0.054	C	A	0.836	3.5E-02	1243
rs9936385	cg00320980	FTO	-0.088	0.042	C	T	0.623	3.5E-02	1242
rs7202877	cg07184465	CTRB1-CTRB2	-0.138	0.066	G	T	0.901	3.5E-02	1243
rs4812829	cg08570691	HNF4A	0.118	0.056	A	G	0.852	3.6E-02	1243
rs1552224	cg07212837	ARAP1,CENTD2	0.114	0.054	C	A	0.836	3.6E-02	1243
rs4812829	cg25136644	HNF4A	0.118	0.056	A	G	0.852	3.6E-02	1243
rs9936385	cg11252555	FTO	-0.087	0.042	C	T	0.623	3.6E-02	1242
rs17106184	cg11983038	FAF1	-0.141	0.067	A	G	0.907	3.6E-02	1236
rs1552224	cg27115863	ARAP1,CENTD2	0.113	0.054	C	A	0.836	3.7E-02	1243
rs9472138	cg25536676	VEGFA	0.093	0.045	T	C	0.712	3.7E-02	1243
rs12571751	cg09185884	ZMIZ1	0.085	0.041	G	A	0.543	3.7E-02	1233
rs944801	cg11851382	NA	0.085	0.041	G	C	0.574	3.7E-02	1219
rs702634	cg07212837	ARL15	0.093	0.045	G	A	0.709	3.7E-02	1213
rs11717195	cg11983038	ADCY5	-0.094	0.045	C	T	0.737	3.7E-02	1236
rs1513272	cg14003143	NA	-0.084	0.040	C	T	0.505	3.7E-02	1231
rs7845219	cg07212837	TP53INP1	0.082	0.039	T	C	0.511	3.8E-02	1229
rs243020	cg26270261	NA	-0.083	0.040	G	A	0.540	3.8E-02	1238
rs10276674	cg27115863	NA	-0.111	0.053	C	T	0.816	3.8E-02	1203

ESM Table 3. (*Continued*)

SNP	CpG	SNP gene	Estimate	SE	EA	OA	EAF	*p*	N
rs77981966	cg04567334	THADA	-0.163	0.079	T	C	0.928	3.8E-02	1206
rs319598	cg16192197	PCBD2	-0.086	0.042	T	C	0.582	3.8E-02	1237
rs1513272	cg07068382	NA	0.084	0.040	C	T	0.505	3.8E-02	1231
rs10788575	cg16575444	PTEN	-0.111	0.054	A	G	0.835	3.8E-02	1236
rs1513272	cg08570691	NA	-0.084	0.040	C	T	0.505	3.9E-02	1231
rs6937795	cg26270261	IL20RA	-0.084	0.040	C	A	0.521	3.9E-02	1223
rs2583941	cg08857797	RPSAP52	0.134	0.065	A	G	0.897	3.9E-02	1240
rs7732130	cg07212837	ZBED3-AS1	0.091	0.044	G	A	0.697	4.0E-02	1207
rs319598	cg08570691	PCBD2	0.085	0.042	T	C	0.582	4.0E-02	1237
rs7845219	cg06039489	TP53INP1	-0.081	0.039	T	C	0.511	4.0E-02	1229
rs1513272	cg24686009	NA	0.083	0.040	C	T	0.505	4.0E-02	1231
rs35720761	cg27374726	THADA	-0.122	0.060	T	C	0.871	4.0E-02	1236
rs17106184	cg00144180	FAF1	0.137	0.067	A	G	0.907	4.1E-02	1236
rs77981966	cg08570691	THADA	-0.161	0.079	T	C	0.928	4.1E-02	1206
rs6937795	cg08945443	IL20RA	-0.083	0.040	C	A	0.521	4.1E-02	1223
rs6937795	cg00989505	IL20RA	-0.083	0.040	C	A	0.521	4.1E-02	1223
rs340874	cg20812370	PROX1	0.083	0.041	T	C	0.577	4.2E-02	1242
rs1387153	cg00574958	MTNR1B	0.089	0.044	T	C	0.707	4.2E-02	1243
rs10842994	cg16575444	KLHDC5	-0.103	0.051	T	C	0.804	4.2E-02	1234
rs10842994	cg25536676	KLHDC5	-0.103	0.051	T	C	0.804	4.2E-02	1234
rs35720761	cg22680424	THADA	-0.121	0.060	T	C	0.871	4.3E-02	1236
rs12427353	cg15560632	HNF1A	0.105	0.052	C	G	0.804	4.3E-02	1201
rs10788575	cg07068382	PTEN	-0.108	0.054	A	G	0.835	4.3E-02	1236
rs319598	cg00162348	PCBD2	-0.084	0.042	T	C	0.582	4.3E-02	1237
rs11717195	cg07212837	ADCY5	-0.091	0.045	C	T	0.737	4.4E-02	1236
rs1974620	cg14003143	NA	-0.080	0.040	C	T	0.528	4.4E-02	1243
rs1359790	cg16765088	SPRY2	0.094	0.047	A	G	0.725	4.4E-02	1216
rs10190052	cg22680424	TMEM18	0.102	0.051	T	C	0.813	4.4E-02	1243
rs77981966	cg22680424	THADA	-0.158	0.079	T	C	0.928	4.4E-02	1206
rs1359790	cg25136644	SPRY2	0.094	0.047	A	G	0.725	4.5E-02	1216
rs1387153	cg16192197	MTNR1B	0.088	0.044	T	C	0.707	4.5E-02	1243
rs2583941	cg00896068	RPSAP52	-0.131	0.065	A	G	0.897	4.5E-02	1240
rs11717195	cg06114363	ADCY5	0.091	0.045	C	T	0.737	4.5E-02	1236
rs10811660	cg20231084	NA	-0.102	0.051	A	G	0.818	4.5E-02	1222
rs1513272	cg27037013	NA	-0.081	0.040	C	T	0.505	4.5E-02	1231
rs6937795	cg20116935	IL20RA	-0.081	0.040	C	A	0.521	4.5E-02	1223

ESM Table 3. (*Continued*)

SNP	CpG	SNP gene	Estimate	SE	EA	OA	EAF	*p*	N
rs1552224	cg27374726	ARAP1,CENTD2	0.109	0.054	C	A	0.836	4.5E-02	1243
rs35261542	cg22680424	CDKAL1	-0.092	0.046	A	C	0.740	4.5E-02	1229
rs4275659	cg01963618	NA	0.090	0.045	T	C	0.702	4.5E-02	1216
rs1496653	cg11376147	NA	-0.096	0.048	G	A	0.782	4.6E-02	1243
rs9936385	cg26766064	FTO	-0.083	0.042	C	T	0.623	4.6E-02	1242
rs1387153	cg01963618	MTNR1B	-0.087	0.044	T	C	0.707	4.6E-02	1243
rs7607980	cg19693031	COBLL1	-0.115	0.058	C	T	0.863	4.6E-02	1242
rs13266634	cg25536676	SLC30A8	0.086	0.043	T	C	0.685	4.7E-02	1243
rs41278853	cg11851382	MTMR3	-0.131	0.066	G	A	0.895	4.7E-02	1242
rs2284219	cg01577083	CRHR2	0.083	0.042	A	G	0.650	4.7E-02	1233
rs1535500	cg07068382	KCNK16	0.078	0.039	T	G	0.522	4.8E-02	1229
rs2284219	cg11983038	CRHR2	-0.083	0.042	A	G	0.650	4.8E-02	1233
rs10190052	cg06500161	TMEM18	0.101	0.051	T	C	0.813	4.8E-02	1243
rs1974620	cg25136644	NA	-0.078	0.040	C	T	0.528	4.9E-02	1243
rs4275659	cg22680424	NA	0.089	0.045	T	C	0.702	4.9E-02	1216
rs12571751	cg20116935	ZMIZ1	0.080	0.041	G	A	0.543	4.9E-02	1233
rs77981966	cg00144180	THADA	-0.155	0.079	T	C	0.928	4.9E-02	1206
rs17106184	cg22680424	FAF1	0.132	0.067	A	G	0.907	4.9E-02	1236
rs780094	cg20812370	GCKR	-0.079	0.040	T	C	0.586	5.0E-02	1243
rs702634	cg01577083	ARL15	0.088	0.045	G	A	0.709	5.0E-02	1213
rs35720761	cg20231084	THADA	-0.117	0.060	T	C	0.871	5.0E-02	1236

Estimate: additive effect of the genotype on a unit change in inverse-normal transformed residuals of DNA methylation, EA: effect allele, OA: other allele, EAF: effect allele frequency, N: sample-size.

ESM Table 4. Methylation quantitative trait loci (mQTL) associated with a subset of the meta-EWAS of T2D CpGs. mQTL were retrieved from the Genetics of DNAm consortium (GoDMC). In total, 39 mQTL proxying 30 of the 58 meta-EWAS CpGs, were used as instruments in the reverse two-sample MR analysis (DNAm → T2D). Associations are ordered by *p*-value (from the smallest to the largest).

SNP	CpG	CpG gene	Cis/Trans	Estimate	SE	EA	OA	EAF	*p*	N
rs11693641	cg00144180	*HDAC4*	Cis	-0.150	0.010	A	C	0.5	3.5E-202	23360
rs113786621	cg00896068	*Open sea*	Cis	-0.340	0.020	T	C	0.08	3.5E-202	25956
rs56261297	cg07212837	*GRINA*	Cis	-0.340	0.010	T	C	0.41	3.5E-202	27738
rs115738369	cg10584271	*ITIH1*	Cis	-1.740	0.030	T	C	0.02	3.5E-202	22070
rs11652574	cg11024682	*SREBF1*	Cis	1.100	0.030	A	G	0.04	3.5E-202	19085
rs11584621	cg12593793	*Open sea*	Cis	-0.060	0.010	A	T	0.21	3.5E-202	25084
rs347903	cg14476101	*PHGDH*	Cis	0.230	0.010	T	C	0.67	3.5E-202	24554
rs750129	cg20231084	*Open sea*	Cis	0.140	0.010	A	G	0.47	3.5E-202	23944
rs55760516	cg20456243	*SPEG*	Cis	-0.120	0.010	A	G	0.67	3.5E-202	27242
rs9309801	cg24512093	*ROBO1*	Cis	-0.110	0.010	T	C	0.34	3.5E-202	27235
rs9831014	cg24512093	*ROBO1*	Cis	0.120	0.010	C	G	0.42	3.5E-202	24994
rs6681644	cg25536676	*DHCR24*	Cis	0.260	0.010	C	G	0.42	3.5E-202	27714
rs13051329	cg27037013	*Open sea*	Cis	0.170	0.010	T	C	0.15	3.5E-202	26837
rs6000773	cg27115863	*Open sea*	Cis	-0.120	0.010	C	G	0.75	3.5E-202	24389
rs9487736	cg16192197	*Open sea*	Cis	0.360	0.010	A	G	0.14	6.3E-188	27726
rs608358	cg14476101	*PHGDH*	Cis	-0.280	0.010	A	C	0.28	5.8E-180	25566
rs1525502	cg10082515	*EIF3IP1*	Cis	-0.250	0.010	T	C	0.41	1.1E-172	24607
rs7496161	cg16765088	*Open sea*	Cis	-0.320	0.010	A	G	0.11	4.7E-122	25984
rs1107095	cg01577083	*Open sea*	Cis	-0.200	0.010	T	C	0.51	7.5E-109	23764
rs7602568	cg27115863	*Open sea*	Trans	0.200	0.010	T	C	0.22	1.0E-85	27625
rs4383852	cg18181703	*SOCS3*	Trans	-0.130	0.010	A	G	0.52	2.3E-56	27746
rs6596785	cg01963618	*LOC285768*	Cis	0.220	0.010	A	G	0.89	2.2E-50	25226
rs1047891	cg08857797	*VPS25*	Trans	-0.140	0.010	A	C	0.32	8.8E-47	24138
rs9525281	cg00896068	*Open sea*	Cis	0.140	0.010	C	G	0.76	3.2E-30	18956
rs174551	cg25536676	*DHCR24*	Trans	0.110	0.010	T	C	0.66	5.1E-30	24653
rs10421294	cg11252555	*RPL13AP5*	Cis	-0.140	0.010	A	G	0.1	2.8E-20	25481
rs540908	cg13178597	*RGS17*	Cis	-0.100	0.010	A	G	0.82	6.4E-18	26442
rs2848634	cg11376147	*SLC43A1*	Cis	0.090	0.010	A	G	0.75	7.9E-18	27749
rs71380866	cg09185884	*KCTD2*	Cis	0.210	0.030	C	G	0.97	8.7E-15	25599
rs62250760	cg10584271	*ITIH1*	Cis	0.050	0.010	T	C	0.35	9.1E-08	25857
rs79365581	cg25536676	*DHCR24*	Cis	0.350	0.070	T	C	0.98	7.8E-07	5834
rs1872614	cg00144180	*HDAC4*	Cis	-0.050	0.010	A	T	0.58	2.2E-06	16512
rs6081870	cg06039489	*C20orf26*	Cis	-0.141	0.010	A	G	0.2685	7.3E-44	24388
rs220182	cg06500161	*ABCG1*	Cis	0.061	0.009	T	C	0.5528	3.5E-202	24474
rs1500138	cg07184465	*SPZ1*	Cis	-0.147	0.009	T	C	0.3522	1.8E-58	25936

ESM Table 4. (*Continued*)

SNP	CpG	CpG gene	Cis/Trans	Estimate	SE	EA	OA	EAF	*p*	N
rs7535757	cg11851382	*PPAP2B (PLPP3)*	Cis	-0.077	0.009	A	G	0.4988	3.5E-202	26658
rs6657798	cg19693031	*TXNIP*	Trans	-0.459	0.010	C	G	0.8024	3.5E-202	27212
rs6732515	cg25741837	*SMYD5*	Cis	0.542	0.032	A	C	0.9767	1.1E-64	22690
rs62148128	cg25741837	*SMYD5*	Cis	0.715	0.021	A	G	0.0613	3.5E-202	20444

Estimate: additive effect of the genotype on a unit change in inverse-normal transformed residuals of DNA methylation as reported by the GoDMC consortium, EA: effect allele, OA: other allele, EAF: effect allele frequency, N: sample-size.

ESM Table 5. Association of mQTL SNPs tagging meta-EWAS CpGs with prevalent T2D using GWAS summary data. GWAS of T2D data was extracted from Mahajan *et al.* 2014 and Wood *et al.* 2016. In total, T2D associations were retrieved for 39 mQTL SNPs proxying 30 meta-EWAS CpGs. Data is ordered by *p*-value (from smallest to largest).

SNP	Exposure	Outcome	T2D GWAS	EA	OA	EAF	Estimate	SE	*p*	N
rs174551*†	cg25536676	T2D	Mahajan et al. 2014	T	C	NA	0.039	0.012	0.011	104377
rs7496161†	cg16765088	T2D	Wood et al. 2016	A	G	0.10	-0.174	0.085	0.034	116171
rs1872614†	cg00144180	T2D	Wood et al. 2016	A	T	0.58	-0.095	0.036	0.037	104810
rs1047891†	cg08857797	T2D	Wood et al. 2016	A	C	0.32	-0.073	0.038	0.042	117775
rs6681644	cg25536676	T2D	Wood et al. 2016	C	G	0.42	0.095	0.033	0.054	117094
rs347903*	cg14476101	T2D	Mahajan et al. 2014	T	C	NA	-0.030	0.015	0.057	107656
rs1500138	cg07184465	T2D	Wood et al. 2016	T	C	0.35	-0.062	0.035	0.072	117682
rs56261297*	cg07212837	T2D	Mahajan et al. 2014	T	C	NA	-0.030	0.012	0.072	96027
rs1525502	cg10082515	T2D	Wood et al. 2016	T	C	0.39	0.095	0.034	0.098	115383
rs7535757	cg11851382	T2D	Wood et al. 2016	A	G	0.49	0.095	0.033	0.100	115683
rs10421294*	cg11252555	T2D	Mahajan et al. 2014	A	G	NA	0.039	0.025	0.110	95397
rs1525502	cg10082515	T2D	Mahajan et al. 2014	T	C	NA	0.020	0.015	0.120	105669
rs1047891*	cg08857797	T2D	Mahajan et al. 2014	A	C	NA	-0.039	0.025	0.150	24243
rs9525281	cg00896068	T2D	Wood et al. 2016	C	G	0.75	0.062	0.046	0.160	109280
rs7535757	cg11851382	T2D	Mahajan et al. 2014	A	G	NA	0.020	0.013	0.170	102682
rs62250760*	cg10584271	T2D	Mahajan et al. 2014	T	C	NA	-0.020	0.015	0.170	106223
rs11693641	cg00144180	T2D	Wood et al. 2016	A	C	0.49	-0.041	0.032	0.180	117061
rs79365581	cg25536676	T2D	Wood et al. 2016	T	C	0.99	0.734	0.540	0.180	117420
rs11693641*	cg00144180	T2D	Mahajan et al. 2014	A	C	NA	-0.020	0.013	0.180	102563
rs2848634	cg11376147	T2D	Mahajan et al. 2014	A	G	NA	-0.020	0.015	0.210	100365
rs56261297	cg07212837	T2D	Wood et al. 2016	T	C	0.41	0.000	0.033	0.270	117775
rs6000773	cg27115863	T2D	Wood et al. 2016	C	G	0.74	0.000	0.042	0.280	116130
rs113786621	cg00896068	T2D	Wood et al. 2016	T	C	0.08	0.095	0.120	0.300	116849
rs6081870	cg06039489	T2D	Wood et al. 2016	A	G	0.27	0.000	0.042	0.300	113116
rs55760516*	cg20456243	T2D	Mahajan et al. 2014	A	G	NA	-0.010	0.013	0.300	104314
rs6732515*	cg25741837	T2D	Mahajan et al. 2014	A	C	NA	0.058	0.067	0.350	63390
rs6732515	cg25741837	T2D	Wood et al. 2016	A	C	0.98	0.288	0.310	0.360	116210
rs10421294	cg11252555	T2D	Wood et al. 2016	A	G	0.09	0.095	0.099	0.370	117526
rs13051329	cg27037013	T2D	Wood et al. 2016	T	C	0.15	-0.051	0.064	0.380	117504
rs174551	cg25536676	T2D	Wood et al. 2016	T	C	0.65	0.000	0.036	0.400	117520
rs55760516	cg20456243	T2D	Wood et al. 2016	A	G	0.68	0.000	0.037	0.400	117775
rs1107095	cg01577083	T2D	Wood et al. 2016	T	C	0.51	0.000	0.033	0.410	110804
rs62250760	cg10584271	T2D	Wood et al. 2016	T	C	0.35	-0.030	0.036	0.420	116430
rs11652574	cg11024682	T2D	Wood et al. 2016	A	G	0.03	-0.198	0.240	0.420	117291

ESM Table 5. (*Continued*)

SNP	Exposure	Outcome	T2D GWAS	EA	OA	EAF	Estimate	SE	*p*	N
rs347903	cg14476101	T2D	Wood et al. 2016	T	C	0.65	0.000	0.037	0.420	111060
rs9309801	cg24512093	T2D	Wood et al. 2016	T	C	0.33	0.000	0.037	0.420	116463
rs4383852	cg18181703	T2D	Wood et al. 2016	A	G	0.52	0.030	0.032	0.420	117641
rs7602568	cg27115863	T2D	Wood et al. 2016	T	C	0.23	-0.030	0.046	0.450	117775
rs540908	cg13178597	T2D	Wood et al. 2016	A	G	0.81	0.000	0.055	0.470	116958
rs6081870	cg06039489	T2D	Mahajan et al. 2014	A	G	NA	0.010	0.015	0.500	106232
rs7496161	cg16765088	T2D	Mahajan et al. 2014	A	G	NA	0.010	0.018	0.510	98254
rs6596785	cg01963618	T2D	Mahajan et al. 2014	A	G	NA	0.010	0.018	0.540	104567
rs9831014	cg24512093	T2D	Wood et al. 2016	C	G	0.42	-0.020	0.034	0.580	115128
rs220182	cg06500161	T2D	Mahajan et al. 2014	T	C	NA	-0.010	0.015	0.620	91284
rs9487736	cg16192197	T2D	Mahajan et al. 2014	A	G	NA	-0.010	0.015	0.630	100236
rs1500138	cg07184465	T2D	Mahajan et al. 2014	T	C	NA	0.010	0.015	0.640	104490
rs2848634	cg11376147	T2D	Wood et al. 2016	A	G	0.75	0.000	0.042	0.680	117587
rs6657798	cg19693031	T2D	Wood et al. 2016	C	G	0.82	0.000	0.056	0.710	116594
rs62148128	cg25741837	T2D	Wood et al. 2016	A	G	0.06	0.095	0.170	0.750	116700
rs750129	cg20231084	T2D	Mahajan et al. 2014	A	G	NA	0.000	0.013	0.770	106230
rs608358	cg14476101	T2D	Wood et al. 2016	A	C	0.28	0.000	0.041	0.780	115429
rs750129	cg20231084	T2D	Wood et al. 2016	A	G	0.47	-0.010	0.036	0.800	96646
rs13051329	cg27037013	T2D	Mahajan et al. 2014	T	C	NA	0.000	0.018	0.800	104328
rs4383852*	cg18181703	T2D	Mahajan et al. 2014	A	G	NA	0.000	0.013	0.810	98320
rs9309801	cg24512093	T2D	Mahajan et al. 2014	T	C	NA	0.000	0.013	0.810	104489
rs11584621	cg12593793	T2D	Wood et al. 2016	A	T	0.21	-0.010	0.051	0.860	113096
rs7602568	cg27115863	T2D	Mahajan et al. 2014	T	C	NA	0.000	0.015	0.910	110135
rs71380866	cg09185884	T2D	Wood et al. 2016	C	G	0.97	0.030	0.300	0.920	117447
rs115738369	cg10584271	T2D	Wood et al. 2016	T	C	0.02	-0.030	0.520	0.950	117430
rs6596785	cg01963618	T2D	Wood et al. 2016	A	G	0.90	0.010	0.097	0.950	115281
rs608358	cg14476101	T2D	Mahajan et al. 2014	A	C	NA	0.000	0.015	0.970	101903
rs220182	cg06500161	T2D	Wood et al. 2016	T	C	0.54	0.000	0.033	1.000	114976

Estimate: effect of the genotype on the log(odds) of prevalent T2D as reported in two previous GWAS (4, 5), EA: effect allele of the outcome, OA: other allele of the outcome, EAF: effect allele frequency of the outcome, N: sample-size of the outcome. In Wood *et al.* 2016 (5), genetic associations with T2D were adjusted for age, sex and the first five within-British principal components. See Supplementary Table 1 for covariates used in Mahajan *et al.* 2014. *mQTL SNPs tagged by an alternative SNP in high LD (r^2 >= 0.6) that was found in the outcome dataset (GWAS of T2D) in MR-Base. Genetic proxies for the mQTL SNPs rs347903, rs10421294, rs1047891, rs174551, rs11693641, rs4383852, rs55760516, rs56261297, rs6732515 and rs62250760, were tagged by the SNPs rs838990, rs4802613, rs715, rs1535, rs1399629,

rs2884013, rs1050816, rs7459603, rs6727270 and rs13314396, respectively, using data from the 1,000 genomes in Europeans. †SNPs associated with T2D with borderline GWAS significance at $p < 0.05$.

ESM Table 6. Results of the reverse two-sample MR (DNAm → T2D) using additional MR methods at the CpG cg25536676 (*DHCR24*). Three mQTL were identified as genetic proxies for this CpG in the GoDMC data set.

Outcome	T2D GWAS	Method	# SNPs	OR (95%CI)	*p*	Het	*p*-Het
T2D	Mahajan et al. 2014	Wald ratio	1	1.43(1.15,1.78)	0.001		
T2D	Wood et al. 2016	IVW	3	1.39(1.08,1.79)	0.011	2.41	0.30
T2D	Wood et al. 2016	MR Egger	3	1.95(0.99,3.85)	0.305	1.15	0.28
T2D	Wood et al. 2016	Weighted median	3	1.38(1.1,1.73)	0.006		
T2D	Wood et al. 2016	Weighted mode	3	1.39(1.1,1.75)	0.107		

IVW: inverse variance-weighted regression. # SNPs: number of single nucleotide polymorphisms used to proxy the CpG cg25536676 (*DHCR24*) and included in the MR analysis. Het: heterogeneity value calculated using the Cochran's Q and Rucker's estimates for the IVW and MR Egger regressions, respectively. *p*-Het: *p* value of the heterogeneity test. Effect estimates are expressed in odds ratios of T2D per increase in inverse normal-transformed residuals of DNA methylation at cg25536676.

ESM Table 7. Comparison of association estimates between the observational and the causal analysis (bidirectional two-sample MR) for 58 CpGs previously reported in a meta-EWAS of prevalent T2D (*p*< 1.3x10^{-5}). Likely causal direction of association was established based on the smallest *p*-value, and on the directional consistency found between the causal and the observational effect estimates for each direction of the 2SMR. *Inconclusive – single direction* if MR data was only available in one direction and *p* > 0.1; *inconclusive-bidirectional* if MR data was available in two directions, but *p* > 0.1 in both cases.

			**Meta-EWAS of T2D (N= 3,248)**		**Forward 2SMR (T2D → DNAm)**		**Reverse 2SMR (DNAm → T2D)**		**Likely causal direction**
CpG	Chr	Gene	Estimate (95%CI)	*p*	Estimate (95%CI)	*P (IVW)*	Estimate (95%CI)	*p (Wald ratio)*	
cg06114363	1	*ZNF683*	-0.01(-0.015,-0.006)	1.37E-06	-0.017(-0.13,0.1)	0.778	NA	NA	Inconclusive - single direction
cg11851382**	1	*PPAP2B(PLPP3)*	-0.008(-0.011,-0.004)	6.42E-06	0.031(-0.09,0.15)	0.603	-1.24(-2.09,-0.4)	0.004	DNAm to T2D
cg12593793	1	*LMNA*	-0.008(-0.011,-0.004)	2.90E-06	0.039(-0.08,0.16)	0.519	0.17(-1.5,1.83)	0.844	T2D to DNAm
cg14476101	1	*PHGDH*	-0.015(-0.021,-0.008)	9.46E-06	-0.008(-0.14,0.12)	0.901	-0.05(-0.18,0.07)	0.398	Inconclusive - bidirectional
cg19693031†	1	*TXNIP*	-0.019(-0.024,-0.014)	8.75E-14	-0.035(-0.15,0.08)	0.555	0.00(-0.24,0.24)	1.000	Inconclusive - bidirectional
cg20812370*	1	*PBX1*	-0.007(-0.009,-0.004)	7.40E-07	-0.184(-0.3,-0.07)	0.002	NA	NA	T2D to DNAm
cg25536676**	1	*DHCR24*	-0.008(-0.011,-0.004)	5.39E-06	0.042(-0.08,0.16)	0.490	0.36(0.14,0.58)	0.001	DNAm to T2D
cg00144180†‡	2	*HDAC4*	0.012(0.008,0.017)	5.64E-08	0.071(-0.05,0.19)	0.256	0.13(-0.03,0.3)	0.115	DNAm to T2D
cg20316538	2	*RUFY4*	-0.005(-0.007,-0.003)	6.11E-06	-0.013(-0.14,0.11)	0.841	NA	NA	Inconclusive - single direction
cg20456243*	2	*SPEG*	-0.007(-0.011,-0.004)	9.99E-06	-0.066(-0.19,0.06)	0.304	0.08(-0.12,0.29)	0.429	T2D to DNAm
cg25741837	2	*SMYD5*	0.009(0.005,0.013)	4.76E-06	0.043(-0.07,0.16)	0.474	0.11(-0.14,0.35)	0.385	DNAm to T2D
cg10584271	3	*ITIH1*	-0.014(-0.019,-0.009)	1.73E-07	-0.068(-0.19,0.06)	0.279	-0.4(-0.98,0.19)	0.187	DNAm to T2D
cg20116935	3	*SEMA3B*	-0.006(-0.009,-0.003)	8.89E-06	-0.047(-0.17,0.07)	0.430	NA	NA	Inconclusive - single direction
cg24512093	3	*ROBO1*	-0.01(-0.013,-0.006)	7.16E-07	-0.036(-0.15,0.08)	0.546	0.00(-0.23,0.23)	1.000	T2D to DNAm
cg07184465	5	*SPZ1*	-0.007(-0.01,-0.004)	7.18E-06	-0.051(-0.17,0.07)	0.403	0.42(-0.05,0.89)	0.077	DNAm to T2D
cg01963618	6	*LOC285768*	-0.008(-0.011,-0.004)	1.55E-06	-0.002(-0.12,0.12)	0.970	0.05(-0.11,0.2)	0.572	T2D to DNAm
cg07068382	6	*MTCH1*	0.01(0.006,0.015)	9.46E-06	0.039(-0.08,0.16)	0.529	NA	NA	Inconclusive - single direction
cg13178597	6	*RGS17*	-0.01(-0.015,-0.006)	8.57E-06	-0.035(-0.15,0.08)	0.560	0.00(-1.08,1.08)	1.000	T2D to DNAm
cg16192197	6	*CCN6*	0.01(0.005,0.014)	3.71E-06	-0.017(-0.14,0.11)	0.784	-0.03(-0.11,0.05)	0.512	DNAm to T2D
cg10082515**	7	*EIF3IP1*	-0.013(-0.019,-0.008)	7.46E-06	-0.046(-0.16,0.07)	0.443	-0.38(-0.65,-0.11)	0.005	DNAm to T2D
cg15560632	7	*LRCH4*	-0.001(-0.001,-0.001)	3.83E-06	-0.038(-0.16,0.08)	0.530	NA	NA	Inconclusive - single direction

ESM Table 7. (*Continued*)

CpG	Chr	Gene	Meta-EWAS of T2D (N= 3,248) Estimate (95%CI)	*p*	Forward 2SMR (T2D → DNAm) Estimate (95%CI)	*p*	Reverse 2SMR (DNAm → T2D) Estimate (95%CI)	*p*	Likely causal direction
cg25136644	7	*ATG9B*	-0.007(-0.01,-0.004)	7.27E-07	-0.09(-0.21,0.03)	0.132	NA	NA	Inconclusive - single direction
cg07212837**	8	*GRINA*	0.006(0.004,0.009)	3.28E-06	-0.069(-0.2,0.06)	0.304	0.09(0.02,0.16)	0.018	DNAm to T2D
cg20154947	8	*PLEC1*	-0.002(-0.003,-0.001)	4.34E-06	0.005(-0.11,0.12)	0.938	NA	NA	Inconclusive - single direction
cg00320980	10	*ADGRA1*	-0.009(-0.013,-0.005)	7.97E-06	-0.045(-0.16,0.07)	0.449	NA	NA	Inconclusive - single direction
cg04567334	10	*CDH23*	-0.006(-0.008,-0.004)	1.67E-07	0.035(-0.09,0.16)	0.567	NA	NA	Inconclusive - single direction
cg08945443	10	*ZMYND17*	0.01(0.006,0.015)	2.64E-06	0.005(-0.12,0.13)	0.939	NA	NA	Inconclusive - single direction
cg19876302	10	*ATE1*	-0.008(-0.011,-0.005)	2.22E-06	0.045(-0.07,0.16)	0.450	NA	NA	Inconclusive - single direction
cg27374726	10	*LINC02681*	-0.009(-0.012,-0.005)	2.32E-06	-0.079(-0.2,0.04)	0.187	NA	NA	Inconclusive - single direction
cg00574958†	11	*CPT1A*	-0.007(-0.01,-0.005)	1.20E-08	0.008(-0.13,0.14)	0.915	NA	NA	Inconclusive - single direction
cg11376147	11	*SLC43A1*	-0.006(-0.008,-0.003)	5.43E-06	-0.045(-0.16,0.07)	0.454	-0.22(-0.55,0.11)	0.187	DNAm to T2D
cg15832662	11	*RTN3*	-0.009(-0.013,-0.005)	8.45E-06	-0.005(-0.14,0.13)	0.945	NA	NA	Inconclusive - single direction
cg20231084	11	*LINC02551*	-0.006(-0.009,-0.003)	8.36E-06	0.01(-0.11,0.13)	0.871	-0.07(-0.58,0.43)	0.780	DNAm to T2D
cg22680424	11	*HCCA2*	0.008(0.005,0.011)	2.16E-06	-0.099(-0.22,0.02)	0.109	NA	NA	Inconclusive - single direction
cg24686009	12	*RAP1B*	-0.002(-0.003,-0.001)	1.19E-06	0.004(-0.11,0.12)	0.942	NA	NA	Inconclusive - single direction
cg24795867	12	*WNT5B*	-0.006(-0.009,-0.004)	2.47E-06	0.045(-0.07,0.16)	0.450	NA	NA	Inconclusive - single direction
cg26270261	12	*KRT4*	-0.006(-0.009,-0.004)	5.68E-07	0.05(-0.07,0.17)	0.418	NA	NA	Inconclusive - single direction
cg00896068	13	*RASA3*	-0.008(-0.011,-0.004)	7.58E-06	-0.077(-0.2,0.05)	0.233	0.11(-0.6,0.81)	0.767	Inconclusive - bidirectional
cg11983038	13	*STARD13*	-0.017(-0.023,-0.01)	7.23E-07	0.039(-0.09,0.16)	0.539	NA	NA	Inconclusive - single direction
cg00989505	14	*MIR299*	-0.004(-0.006,-0.002)	9.33E-06	-0.034(-0.16,0.09)	0.601	NA	NA	Inconclusive - single direction
cg16765088**†	15	*SYNM*	-0.011(-0.014,-0.007)	5.50E-10	-0.079(-0.2,0.04)	0.188	0.54(0.02,1.07)	0.040	DNAm to T2D
cg00162348	16	*RNF40*	-0.002(-0.003,-0.001)	6.64E-06	0.018(-0.1,0.14)	0.766	NA	NA	Inconclusive - single direction
cg01577083*	16	*RBFOX1*	-0.011(-0.016,-0.006)	7.93E-06	-0.151(-0.28,-0.02)	0.023	0.00(-0.32,0.32)	1.000	T2D to DNAm
cg16575444	16	*CX3CL1*	-0.006(-0.008,-0.004)	6.83E-07	0.01(-0.11,0.13)	0.862	NA	NA	Inconclusive - single direction
cg08857797	17	*VPS25*	0.009(0.005,0.012)	2.28E-06	-0.059(-0.19,0.07)	0.377	0.52(-0.01,1.05)	0.056	DNAm to T2D
cg09185884	17	*KCTD2*	0.011(0.006,0.015)	2.33E-06	0.001(-0.12,0.12)	0.989	0.15(-2.65,2.95)	0.919	Inconclusive - bidirectional

ESM Table 7. (*Continued*)

CpG	Chr	Gene	Meta-EWAS of T2D (N= 3,248) Estimate (95%CI)	*p*	Forward 2SMR (T2D → DNAm) Estimate (95%CI)	*p*	Reverse 2SMR (DNAm → T2D) Estimate (95%CI)	*p*	Likely causal direction
cg11024682	17	*SREBF1*	0.008(0.005,0.011)	1.33E-06	0.054(-0.07,0.18)	0.414	-0.18(-0.61,0.25)	0.408	T2D to DNAm
cg14284506	17	*LGALS9*	-0.005(-0.007,-0.003)	7.31E-06	-0.033(-0.17,0.11)	0.640	NA	NA	Inconclusive - single direction
cg18181703	17	*SOCS3*	-0.01(-0.015,-0.006)	6.20E-06	0.075(-0.04,0.19)	0.217	-0.23(-0.72,0.25)	0.341	DNAm to T2D
cg26766064*	17	*MIR657*	-0.007(-0.009,-0.004)	5.17E-06	-0.1(-0.22,0.02)	0.096	NA	NA	T2D to DNAm
cg08570691	19	*RPL13AP5*	-0.008(-0.012,-0.005)	2.78E-06	-0.07(-0.21,0.07)	0.327	NA	NA	Inconclusive - single direction
cg11252555	19	*RPL13AP5*	-0.008(-0.011,-0.004)	7.44E-06	-0.054(-0.17,0.06)	0.370	-0.28(-0.62,0.06)	0.110	DNAm to T2D
cg24704287†	19	*MIR23A*	-0.011(-0.015,-0.007)	2.34E-08	-0.009(-0.13,0.11)	0.877	NA	NA	Inconclusive - single direction
cg06039489	20	*C20orf26*	0.016(0.009,0.022)	2.71E-06	-0.105(-0.22,0.01)	0.080	-0.07(-0.28,0.14)	0.512	T2D to DNAm
cg14003143	20	*SGK2*	-0.006(-0.008,-0.003)	4.12E-06	0.024(-0.1,0.15)	0.710	NA	NA	Inconclusive - single direction
cg06500161†	21	*ABCG1*	0.013(0.009,0.017)	2.34E-11	-0.067(-0.18,0.05)	0.263	-0.16(-0.65,0.32)	0.512	Inconclusive - bidirectional
cg27037013	21	*LINC00649*	-0.015(-0.021,-0.009)	2.90E-06	-0.044(-0.16,0.07)	0.465	-0.30(-1.04,0.44)	0.423	T2D to DNAm
cg27115863	22	*CARD10*	-0.011(-0.015,-0.006)	2.41E-06	-0.078(-0.2,0.04)	0.207	0.00(-0.15,0.15)	1.000	T2D to DNAm

Meta-EWAS of T2D: meta-analysis of epigenome-wide association studies of prevalent T2D (N=3,428). 2SMR: Two-sample Mendelian randomisation. DNAm: DNA methylation. Associations were considered significant at $p < 1.33 \times 10^{-7}$ in the meta-EWAS of T2D, $p < 0.001$ in the forward 2SMR, and $p < 0.002$ in the reverse 2SMR analysis. * CpGs identified with borderline significance in the forward 2SMR. ** CpGs identified with significance or borderline significance in the reverse 2SMR. †CpGs previously reported with epigenome-wide significance in the meta-EWAS of T2D.‡meta-EWAS CpG analyzed bidirectionally in the 2SMR and observed with consistent direction of association between the causal and the observational analysis. NA: missing data.

ESM Figure 1. Summary plots of the forward two-sample Mendelian randomisation at the CpG cg20812370 (*PBX1*), previously detected in a meta-EWAS of prevalent T2D.

A) Scatter plot depicting the effect of 62 SNPs as individual instruments on T2D (x-axis) and on DNA methylation at cg20812370 (*PBX1*) (y-axis). The fitted regression lines represent the combined effect of these SNPs as a single instrument on DNAm at cg20812370 (*PBX1*). The color of the fitted line refers to the specific MR method used. A negative slope was common to all methods, suggesting that T2D was associated with a decrease in inverse-normal transformed residuals of methylation at cg20812370 (*PBX1*). Highlighted in the plot were the SNPs rs10811660 and rs7903146 that we identified with suggestive heterogeneous effects on DNAm at cg20812370. B) Funnel plot showing the causal effect of each SNP (x-axis) against the inverse of the standard error (SE) of the combined causal effect (y-axis). Some asymmetry in the funnel plot was identified due to the extreme negative effect of SNPs rs319598 and rs1359790 on methylation at cg20812370 (*PBX1*), suggesting horizontal pleiotropy. C) Forest plot illustrating the mean causal effect of each T2D SNP on methylation at cg20812370 (*PBX1*). Mean causal effect is depicted by the black point, with the surrounding line corresponding to the 95% confidence interval (95% CI). At the bottom of the plot, the horizontal red lines show the mean and 95% CI of the meta-analyzed causal effect across SNPs using one of five different MR methods. Effect estimates crossing the vertical dashed line set at "0", or the line of null associations, indicate no evidence of causality for the single SNP, or the combined causal effect. D) Leave-one-out sensitivity analysis showing the total effect of T2D on methylation at cg20812370 (*PBX1*) after sequentially excluding one SNP at a time from the analysis. This analysis helped to identify how robust was the estimate to the effect of individual SNPs, and if the total causal effect was driven by a single instrument. For this analysis, none of the SNPs was driving alone the total causal estimate at cg20812370 (*PBX1*), and the combined effect using the IVW estimate suggested strong evidence for a negative effect of T2D on methylation at the CpG in *PBX1*.

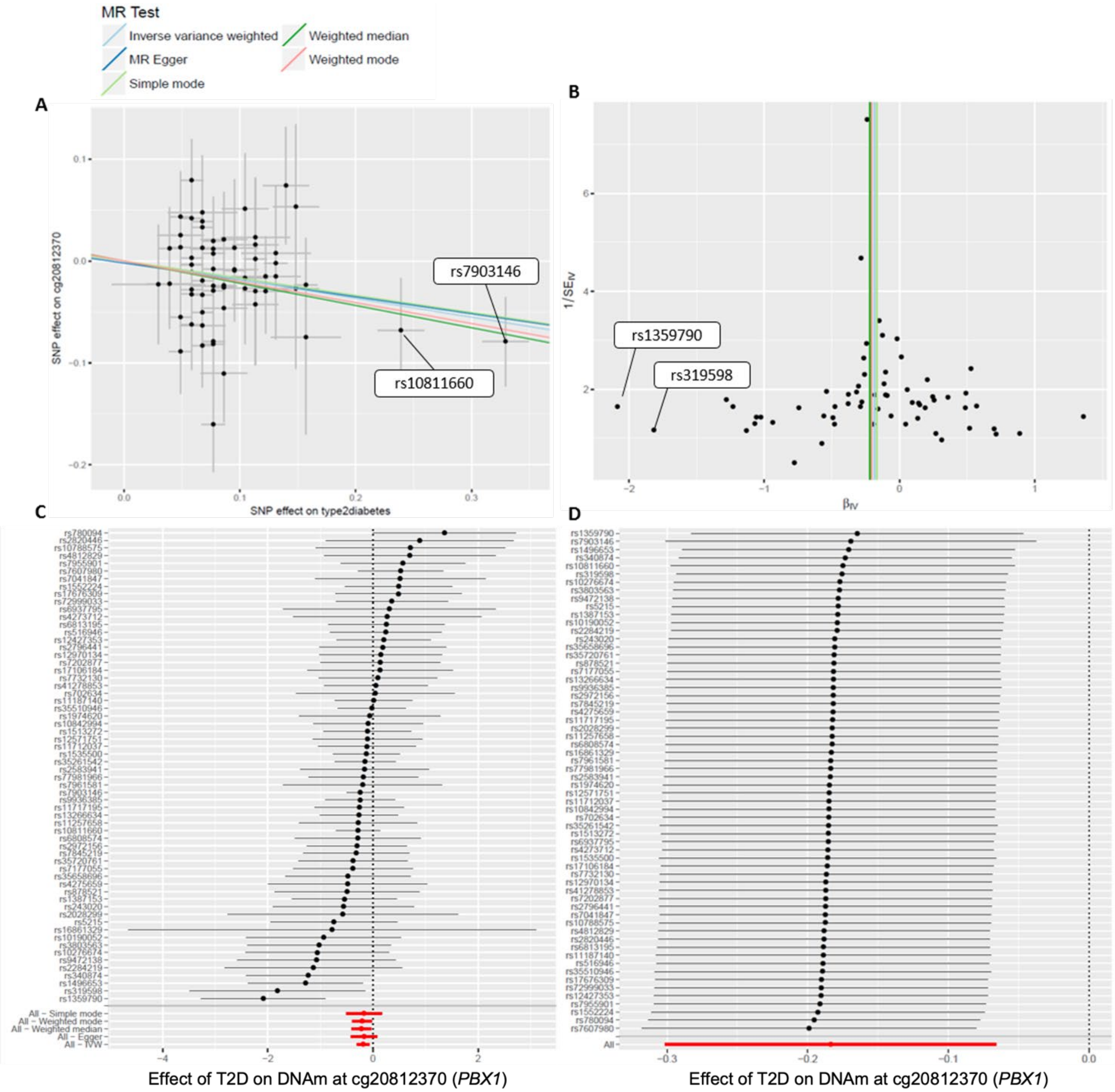
MR Test
Inverse variance weighted
Weighted median
MR Egger
Weighted mode
Simple mode
A
B
SNP effect on cg20812370
SNP effect on type2diabetes
rs7903146
rs10811660
1/SE$_{IV}$
β$_{IV}$
rs1359790
rs319598
C
D
Effect of T2D on DNAm at cg20812370 (*PBX1*)
Effect of T2D on DNAm at cg20812370 (*PBX1*)

ESM Figure 2. Summary plots of the forward two-sample Mendelian randomisation at the CpG cg01577083 (*RBFOX1*), previously detected in a meta-EWAS of prevalent T2D.

A) Scatter plot depicting the effect of 62 SNPs as individual instruments on T2D (x-axis) and on DNA methylation at cg01577083 (*RBFOX1*) (y-axis). The fitted regression lines represent the combined effect of these SNPs as a single instrument on DNAm at cg01577083. The color of the fitted line refers to the specific MR method used. A negative slope was common to all methods, suggesting that T2D was negatively associated with an increase in inverse-normal transformed residuals of methylation at cg01577083. Highlighted in the plot were the SNPs rs10811660 and rs7903146 that we identified with suggestive heterogeneous effects on DNAm at cg01577083. B) Funnel plot showing the causal effect of each SNP (x-axis) against the inverse of the standard error (SE) of the combined causal effect (y-axis). C) Forest plot illustrating the mean causal effect of each T2D SNP on methylation at cg01577083. Mean causal effect is depicted by the black point, with the surrounding line corresponding to the 95% confidence interval (95% CI). At the bottom of the plot, the horizontal red lines show the mean and 95% CI of the meta-analyzed causal effect across SNPs using one of five different MR methods. Effect estimates crossing the vertical dashed line set at "0", or the line of null associations, indicate no evidence of causality for the single SNP, or the combined causal effect. D) Leave-one-out sensitivity analysis showing the total effect of T2D on methylation at cg01577083 after sequentially excluding one SNP at a time from the analysis. For this analysis, none of the SNPs was driving alone the total causal estimate at cg01577083, and the combined effect using the IVW estimate suggested strong evidence for a negative effect of T2D on methylation at this CpG.

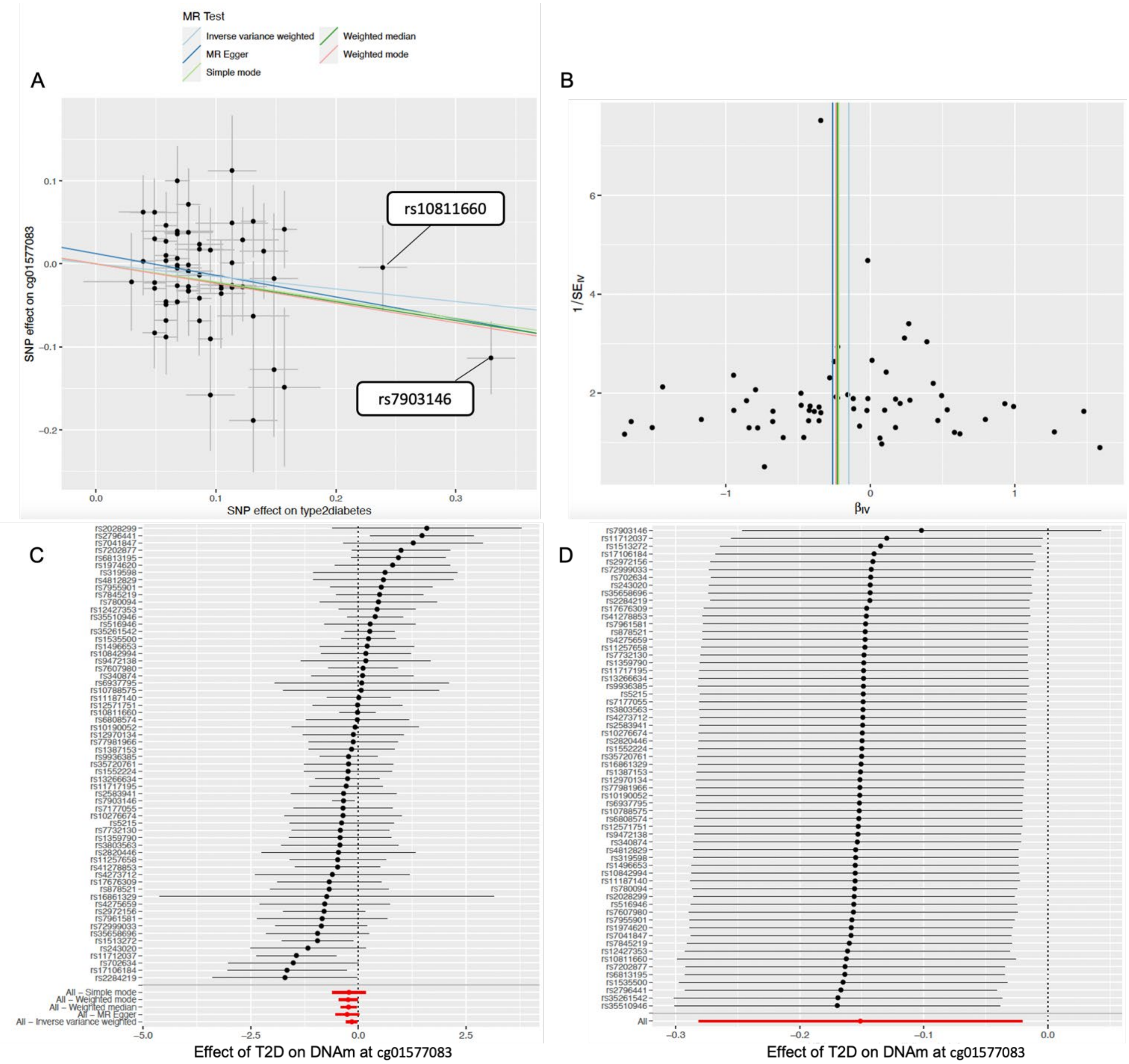

MR Test
Inverse variance weighted
MR Egger
Simple mode
Weighted median
Weighted mode
A
B
C
D
SNP effect on cg01577083
SNP effect on type2diabetes
rs10811660
rs7903146
1/SE$_{IV}$
β$_{IV}$
All – Simple mode
All – Weighted mode
All – Weighted median
All – MR Egger
All – Inverse variance weighted
All
Effect of T2D on DNAm at cg01577083
Effect of T2D on DNAm at cg01577083

ESM Figure 3. Forest plot showing enrichment for outcomes listed in the EWAS catalog based on CpGs detected previously in a meta-EWAS of T2D that were analyzed using a bidirectional two-sample MR (2SMR). Results of the enrichment analysis are shown based on the likely direction of association between meta-EWAS CpGs and T2D according to results of the bidirectional 2SMR.

CpGs identified as secondary to the effects of T2D (forward 2SMR) (n=12)

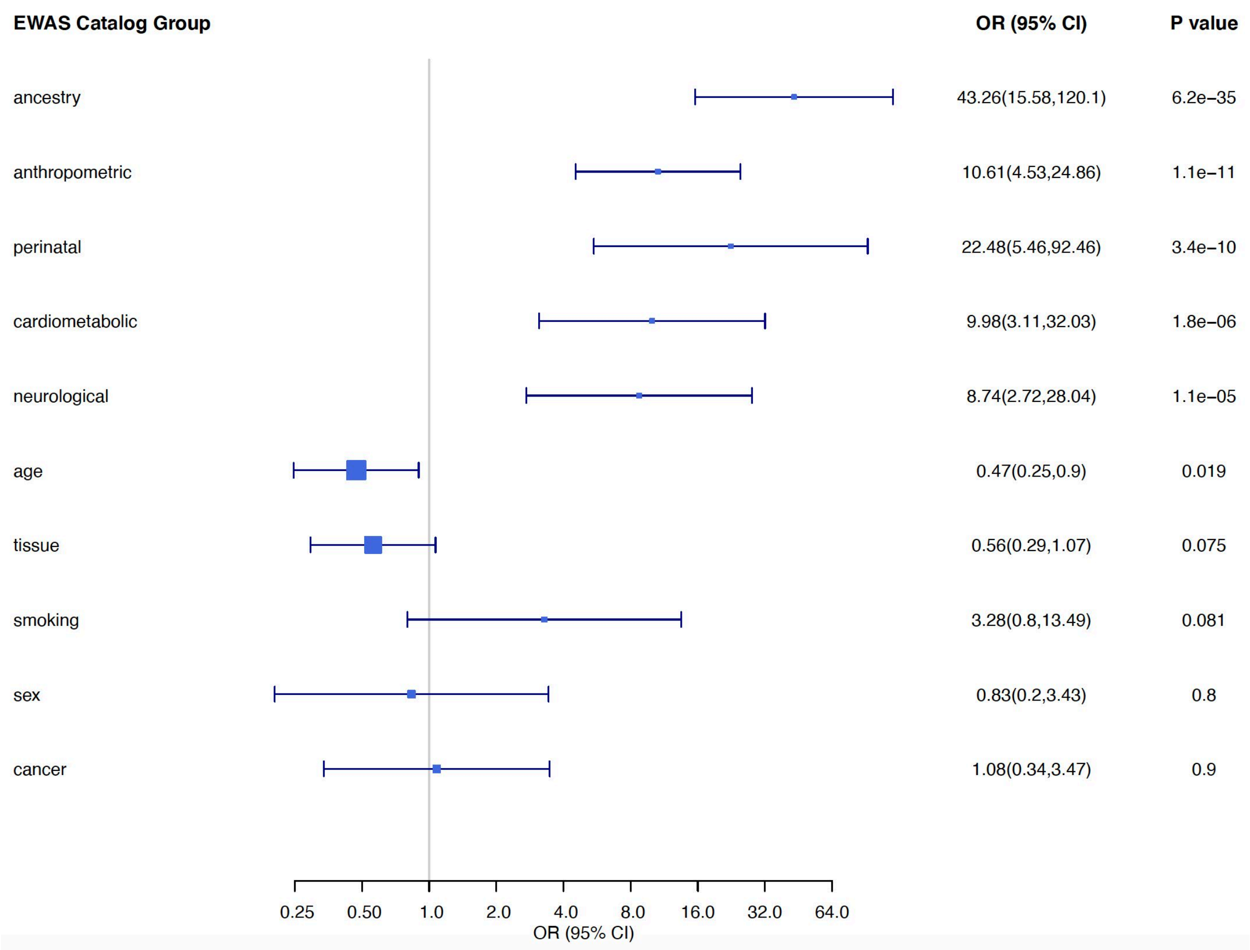

CpGs identified with a likely causal effect on prevalent T2D (reverse 2SMR) (n=15)

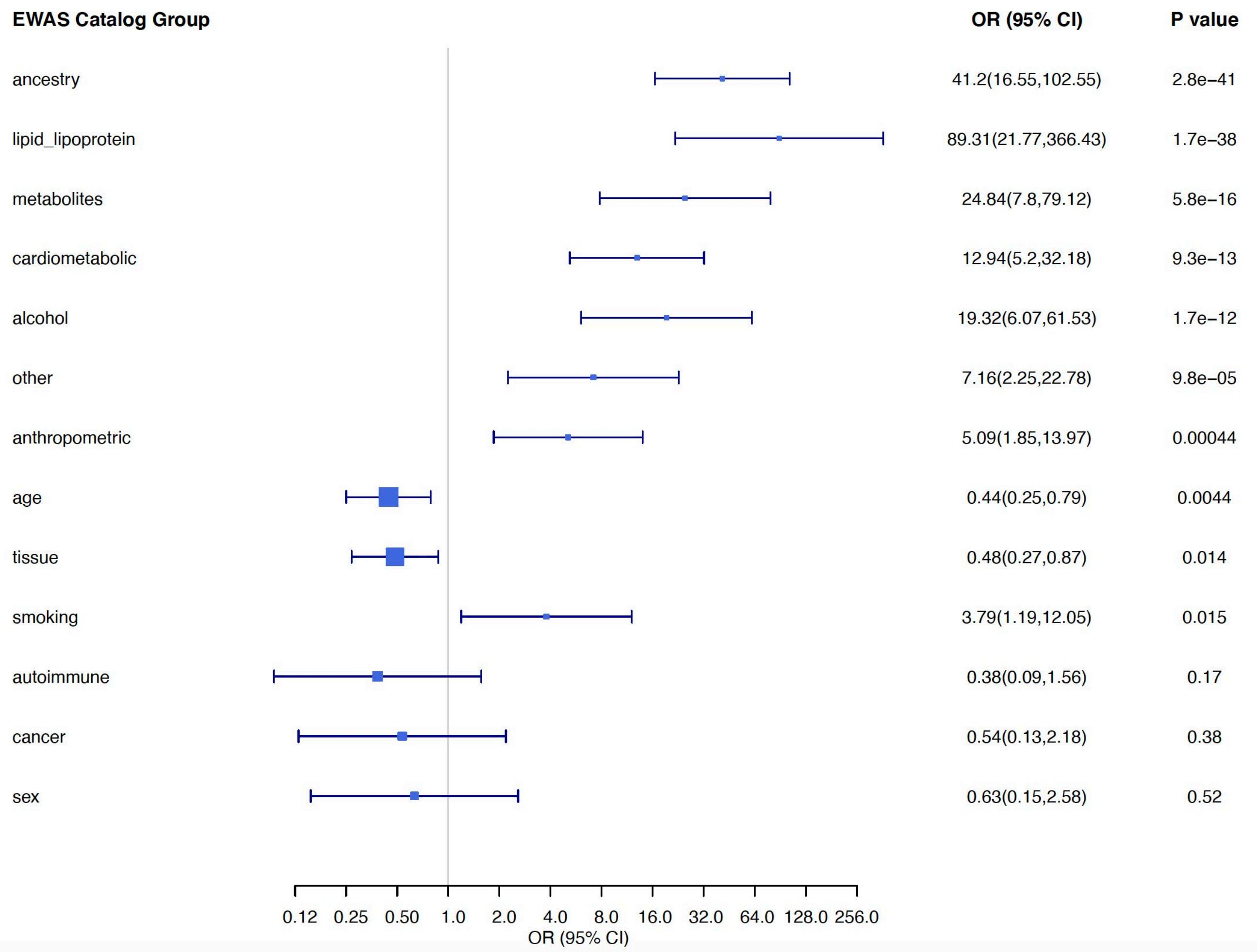

CpGs with inconclusive direction of association with prevalent T2D based on bidirectional 2SMR data (n=31)

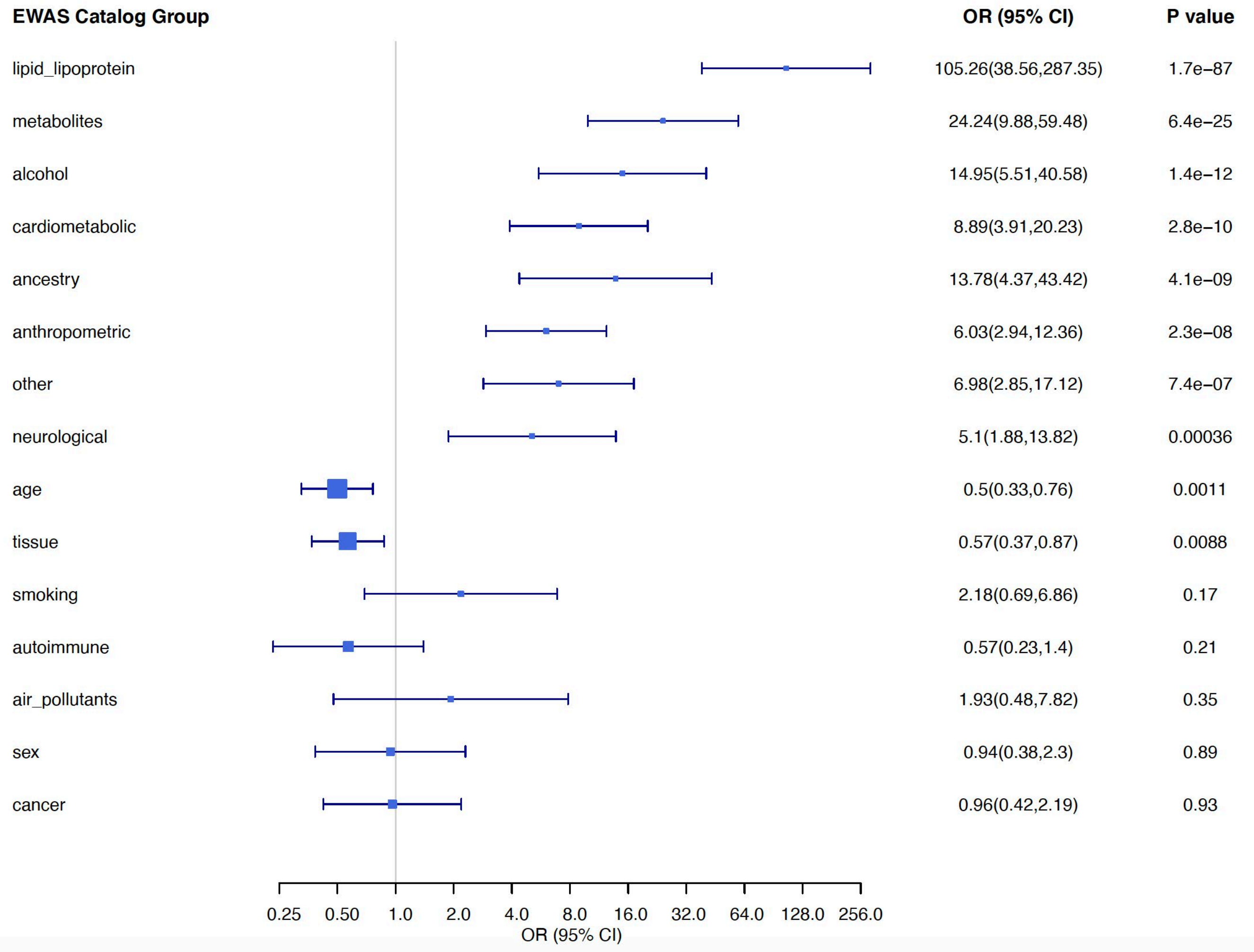

Ancestry: ethnicity. **Anthropometric:** bmi, body_mass_index, waist_circumference, arm_circumference, head_circumference, hip_circumference, fat, mass, weight, height, skinfold, waist, bone_mineral_density, head, hip, leg, pelvis, ribs, spine, total_body, trunk, alpha_neck_angle__hip_measurement, theta_neck_angle__hip_measurement, arm_area and arm_bone_mineral_content. **Autoimmune:** rheumatoid, ulcerative_colitis, lupus, crohn, inflammatory_bowel_disease, atopy, graves, psoriasis, multiple_sclerosis, cow_milk_allergy. **Perinatal:** perinatal, birth_weight, birthweight, maternal_underweight, plasma_folate, prenatal, pregnancy, preterm_birth, season_of_birth, breastfeeding, utero, fetal_intolerance_of_labor, Starling-PS_maternal_serum, gestational_weight_gain, parity and fetal_brain_development. **Cardiometabolic:** blood_pressure, diabetes, chronic_kidney_disease, atrial_fibrillation, ischaemic_stroke, hypertension,

myocardial_infarction, coronary_heart_disease, obesity, hepatic_fat, statin_use, creactive, c-reactive_protein, insulin, glucose, homair, resistin, hba1c, adiponectin, leptin, liver_fat, proinsulin, arterial_distensibility, common_carotid_intima-media_thickness and pulse_rate. **Lipid lipoproteins:** triglycerides, hdl, highdensity_lipoprotein, lipemia, cholesterol, lipoprotein, ldl, vldl, idl, phospholipids, lp_a, concentration_of_idl_particles, total_lipids_in_idl and total_phosphoglycerides. **Neurological:** dementia, schizophrenia, palsy, alzheimer, depressive_disorder, depressive_symptoms, attention_deficit_hyperactivity_disorder, wellbeing, amyloid_plaques, depression, cognitive, personality_disorder, tic_disorders, aggressive_behaviour, infant_attention, cortical, stress, anxiety, neurobehavioural_scale, seizures, conduct_problems, parkinson, social_communication_deficits, hippocampus_volume, thalamus_volume, antidepressant_use, response_to_antidepressants, apolipoprotein and apoe. **"Other"** correspond to traits not included in age, tissue, smoking, alcohol, sex, ancestry, cancer, autoimmune, infection, cardiometabolic, perinatal, lung, lipid_lipoprotein, anthropometric, neurological, socioeconomic position, social_adversity, diet_environment, air_pollutants, metabolites and miRNA. **Tissue:** tissue.

REFERENCES

1. Fraser A, Macdonald-Wallis C, Tilling K, Boyd A, Golding J, Davey Smith G, et al. Cohort Profile: the Avon Longitudinal Study of Parents and Children: ALSPAC mothers cohort. Int J Epidemiol. 2013;42(1):97-110.
2. Boyd A, Golding J, Macleod J, Lawlor DA, Fraser A, Henderson J, et al. Cohort Profile: the 'children of the 90s'--the index offspring of the Avon Longitudinal Study of Parents and Children. Int J Epidemiol. 2013;42(1):111-27.
3. Min JL, Hemani G, Hannon E, Dekkers KF, Castillo-Fernandez J, Luijk R, et al. Genomic and phenotypic insights from an atlas of genetic effects on DNA methylation. Nat Genet. 2021;53(9):1311-21.
4. Mahajan A, Go MJ, Zhang W, Below JE, Gaulton KJ, Ferreira T, et al. Genome-wide trans-ancestry meta-analysis provides insight into the genetic architecture of type 2 diabetes susceptibility. Nat Genet. 2014;46(3):234-44.
5. Wood AR, Tyrrell J, Beaumont R, Jones SE, Tuke MA, Ruth KS, et al. Variants in the FTO and CDKAL1 loci have recessive effects on risk of obesity and type 2 diabetes, respectively. Diabetologia. 2016;59(6):1214-21.
6. Davies NM, Holmes MV, Davey Smith G. Reading Mendelian randomisation studies: a guide, glossary, and checklist for clinicians. BMJ. 2018;362:k601.
7. Hemani G, Zheng J, Elsworth B, Wade KH, Haberland V, Baird D, et al. The MR-Base platform supports systematic causal inference across the human phenome. Elife. 2018;7:e34408.

8. Zheng J, Baird D, Borges MC, Bowden J, Hemani G, Haycock P, et al. Recent Developments in Mendelian Randomization Studies. Curr Epidemiol Rep. 2017;4(4):330-45.
9. Caramaschi D, Sharp GC, Nohr EA, Berryman K, Lewis SJ, Davey Smith G, et al. Exploring a causal role of DNA methylation in the relationship between maternal vitamin B12 during pregnancy and child's IQ at age 8, cognitive performance and educational attainment: a two-step Mendelian randomization study. Hum Mol Genet. 2017;26(15):3001-13.
10. Naeem H, Wong NC, Chatterton Z, Hong MKH, Pedersen JS, Corcoran NM, et al. Reducing the risk of false discovery enabling identification of biologically significant genome-wide methylation status using the HumanMethylation450 array. BMC Genomics. 2014;15(1):51.
11. Zeilinger S, Kühnel B, Klopp N, Baurecht H, Kleinschmidt A, Gieger C, et al. Tobacco smoking leads to extensive genome-wide changes in DNA methylation. PLoS One. 2013;8(5):e63812.
12. Elliott HR, Tillin T, McArdle WL, Ho K, Duggirala A, Frayling TM, et al. Differences in smoking associated DNA methylation patterns in South Asians and Europeans. Clin Epigenetics. 2014;6(1):4.
13. van Buuren S, Groothuis-Oudshoorn K. mice: Multivariate Imputation by Chained Equations in R. Journal of Statistical Software. 2011;45(3):1 - 67.
